# *Arabidopsis* PHOSPHATE TRANSPORTER1 genes *PHT1;8* and *PHT1;9* are involved in root-to-shoot translocation of orthophosphate

**DOI:** 10.1186/s12870-014-0334-z

**Published:** 2014-11-27

**Authors:** Hazel R Lapis-Gaza, Ricarda Jost, Patrick M Finnegan

**Affiliations:** School of Plant Biology, University of Western Australia, 35 Stirling Highway, Crawley (Perth), WA 6009 Australia; Institute of Agriculture, University of Western Australia, 35 Stirling Highway, Crawley (Perth), WA 6009 Australia

**Keywords:** Phosphate transporters, *Arabidopsis*, Gene expression, Local signaling, Systemic signaling

## Abstract

**Background:**

In plants, the uptake from soil and intercellular transport of inorganic phosphate (Pi) is mediated by the PHT1 family of membrane-spanning proton : Pi symporters. The *Arabidopsis thaliana AtPHT1* gene family comprises nine putative high-affinity Pi transporters. While AtPHT1;1 to AtPHT1;4 are involved in Pi acquisition from the rhizosphere, the role of the remaining transporters is less clear.

**Results:**

Pi uptake and tissue accumulation studies in *AtPHT1;8* and *AtPHT1;9* knock-out mutants compared to wild-type plants showed that both transporters are involved in the translocation of Pi from the root to the shoot. Upon inactivation of *AtPHT1;9*, changes in the transcript profiles of several genes that respond to plant phosphorus (P) status indicated a possible role in the regulation of systemic signaling of P status within the plant. Potential genetic interactions were found among PHT1 transporters, as the transcript profile of *AtPHT1;5* and *AtPHT1;7* was altered in the absence of AtPHT1;8, and the transcript profile of *AtPHT1;7* was altered in the *Atpht1;9* mutant. These results indicate that AtPHT1;8 and AtPHT1;9 translocate Pi from the root to the shoot, but not from the soil solution into the root.

**Conclusion:**

AtPHT1;8 and AtPHT1;9 are likely to act sequentially in the interior of the plant during the root-to-shoot translocation of Pi, and play a more complex role in the acclimation of *A. thaliana* to changes in Pi supply than was previously thought.

**Electronic supplementary material:**

The online version of this article (doi:10.1186/s12870-014-0334-z) contains supplementary material, which is available to authorized users.

## Background

Phosphorus (P) is a major essential nutrient for plant growth, development and reproduction. Plants acquire P from the soil in its most oxidized inorganic form, phosphate (Pi) [1]. The uptake of Pi into the plant occurs against a steep electrochemical gradient. While the concentration of Pi in the soil solution is generally less than 2 μM, the Pi concentrations within plant tissues can be greater than 10 mM [2]. However, cytosolic Pi concentrations are tightly controlled, rarely exceeding 60–80 μM Pi [3]. Pi uptake from the soil and transport within the plant against this concentration gradient is mediated by Pi transporters. The first eukaryotic Pi transporter protein to be described was the PHO84p H^+^ : Pi symporter in yeast [4], followed by plant homologs [5,6]. From the numerous plant sequences now available, four PHOSPHATE TRANSPORTER (PHT) families are recognised: PHT1 (plasma membrane), PHT2 (plastid inner envelope), PHT3 (mitochondrial inner membrane) and PHT4 (mostly plastid envelope and one Golgi-localized transporter) [7,8].

The *Arabidopsis* AtPHT1 family has nine members. The family is composed of several high-affinity Pi transporters having K_m_ values in the range of 2.5 μM to 12.3 μM [9] and other members that may have lower affinities for Pi [10,11]. Transcripts from most of the *AtPHT1* genes are detected in both roots and shoots [12-15], while *AtPHT1;6* transcripts are most abundant in flowers [12]. Transcripts from all *AtPHT1* genes except *AtPHT1;6* accumulate upon Pi starvation [16]. Transcriptional regulation of *AtPHT1* expression seems to be mainly controlled by the internal P status [13,15,17,18]. Sugars and cytokinins can also direct the expression of some *AtPHT1* family members [19].

Several strategies have evolved in plants that help them acclimate to variation in Pi availability, including the modulation of *PHT1* gene expression. The deployment of these strategies is modulated by local and systemic signaling networks. The best characterized systemic signaling module involved in the responses to changes in Pi supply includes the phloem-mobile microRNA Atmir399d, its target gene *AtPHO2* and a family of regulatory, non-coding RNAs encoded by the *AtIPS1* and *AtAT4* genes [20,21]. These functions form a circuit where AtPHO2 activity in the root*,* which mediates the ubiquitination of AtPHT1 proteins in the post-endoplasmic reticulum compartment [21], is modulated by Atmir399d as the shoot experiences variations in P levels [14,22]. The activity of Atmir399d in silencing AtPHO2 transcripts is itself antagonistically modulated by At*IPS1* and *AtAT4* transcripts during prolonged periods of Pi starvation [23,24]. On the other hand, local signaling networks control many of the characteristic changes in root system architecture that accompany changes in Pi availability. Thibaud et al. [18] identified a set of genes that are induced by local signaling networks during Pi starvation. These genes include the ethylene-responsive *AtERF1* transcription factor gene, the metalloproteinase *At2-MMP* gene, the jasmonate-inducible *AtGSTU12* and *AtLOX4* genes and the *AtWRKY75* transcription factor gene, which encodes a modulator of both the Pi-starvation response and root development [25].

Functional characterization of *AtPHT1;1* and *AtPHT1;4* validated their roles in Pi acquisition from the soil solution under both Pi-sufficient and Pi-deficient growth conditions [26]. *AtPHT1;5* plays a role in translocating Pi from source to sink organs [27]. Analysis of a *Atpht1;9–1* mutant and *pht1;8/pht1;9* silencing lines suggested a role for AtPHT1;9 and AtPHT1;8 in Pi acquisition at the root-soil interface during prolonged Pi limitation [28]. However, based on the increased transcript abundance from these two *AtPHT1* genes in the *pho2* mutant [22], we hypothesize that AtPHT1;8 and AtPHT1;9 each have a role in translocating Pi from the root to the shoot. In this study we examined the physiological functions of AtPHT1;8 and AtPHT1;9 by characterizing their transcriptional regulation and the phenotypes of corresponding T-DNA insertion mutants in response to changes in Pi supply. Genetic interactions within the *AtPHT1* gene family were also examined by analyzing the transcript patterns of its members in each mutant in response to Pi availability. Furthermore, the placement of these two *AtPHT1* gene functions within the plant response to variations in Pi supply was determined by analyzing the transcript patterns of several genes associated with systemic and local signaling networks in each mutant.

## Results

### Transcripts from individual *AtPHT1* genes responded differentially to Pi deprivation and re-supply

**Figure 1:**
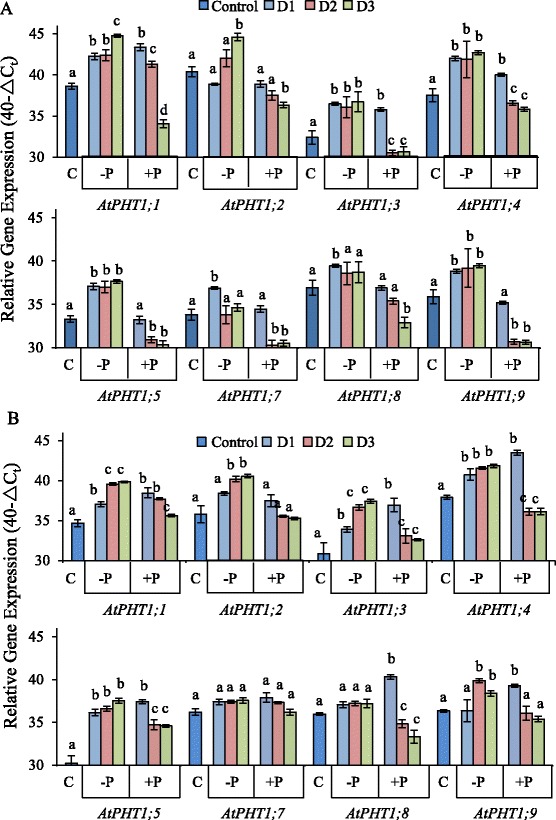
**Responsiveness of** ***AtPHT1*** **transcript levels to Pi supply.** The relative abundance of *AtPHT1* transcripts in roots **(A)** and shoots **(B)** of 42-day-old *Arabidopsis* plants that were deprived of Pi for 12 days followed by Pi resupply in hydroponics is shown. Transcript abundance was measured by qRT-PCR and expressed as 40-∆C_t_, a log2 measure of the ratio of the transcript amount from the target gene to the average transcript amount from a set of reference genes (see Method). Plants were grown in nutrient solution containing 250 μM Pi for 30 d, transferred to solution without Pi for 12 d to deplete internal P pools, and then transferred to a solution containing either no added Pi (−P) or 250 μM Pi (+P). Tissues were harvested 1, 2 or 3 d (D1, D2, D3) after the start of treatments. Control **(C)** plants were continuously supplied with 250 μM Pi and were harvested at D1. Data points are means ± S.D. (n = 3 biological replicates of 12 plants each). Different letters indicate significantly different means (P <0.05) between the control and treatment according to one-way ANOVA and Tukey’s Multiple Comparison of Means. The –P plants and + P plants were statistically analysed as separate groups.

The remodeling kinetics of the *AtPHT1* transcript pool in response to both P depletion and Pi re-supply were examined in well-established *Arabidopsis* plants prior to inflorescence emergence. In this and the following experiments, whenever Pi was supplied, the supply was set to be sufficient for non-limited growth, without being luxuriant. Wild-type (WT) plants were grown hydroponically and then deprived of Pi for 12 days until the leaves began to accumulate anthocyanins (Additional file 1: Figure S1A), a visible indication that the tissues were beginning to experience P depletion. At this time, plants were transferred to nutrient solution containing either no added Pi (P-deprived) or added Pi (Pi re-supply). Quantitative PCR (qPCR) was used to measure transcript abundance for eight of the nine members of the *AtPHT1* gene family in root and shoot tissues over the next three days (Figure 1). *AtPHT1;6* was excluded from the analysis because of its low transcript abundance in roots and shoots [15]. Transcript abundance was normalized to the average transcript abundance for a set of reference genes [29,30]. To conservatively identify genes whose transcript patterns changed with Pi availability, only differences in the 40-ΔC_t_ value of greater than two were considered, corresponding to a four-fold difference in transcript abundance [31].

In *Arabidopsis* roots, P depletion by growth in the absence of a Pi supply for 13 d resulted in a four-fold to 16-fold greater transcript abundance for all the *AtPHT1* genes tested (P ≤0.05), except for *AtPHT1;2*, when compared to control plants continuously supplied with Pi (Figure 1A, *cf.* D1)**.** This is in general agreement with what has previously been observed [13,15,32]. The abundance of *AtPHT1;7* and *AtPHT1;8* transcripts were eight-fold higher after 13 d Pi depletion compared to the control plants, but the abundance of these transcripts was lower at D2 and D3, being indistinguishable in abundance to these transcripts in the control plants under continuous Pi supply.

After 1 d of Pi re-supply to Pi-deprived plants, transcript abundance for most of the *AtPHT1* genes tested in roots was similar to that in the control plants (Figure 1A). The *exceptions* were *AtPHT1;1, AtPHT1;3* and *AtPHT1;4.* The repression of *AtPHT1* transcript abundance by day 2 of Pi re-supply was generally stronger than after day 1. Transcripts from *AtPHT1;3, AtPHT1;4*, *AtPHT1;5, AtPHT1;7* and *AtPHT1;9* were repressed to their lowest levels at this time point. Transcripts from *AtPHT1;1*, *AtPHT1;2* and *AtPHT1;8* were repressed to their lowest levels at day 3 of Pi re-supply (Figure 1A). Interestingly, 3 d of Pi re-supply repressed the abundance of *AtPHT1* transcripts to levels below those present in control plants that had been continuously supplied with Pi for the entire experiment even though the root Pi concentration was the same as in the control plants (Additional file 1: Figure S1B).

The general trends of transcript accumulation in the shoots of Pi-deprived plants were similar to those found in the roots (Figure 1B), except for *AtPHT1;7* and *AtPHT1;8*, where transcript abundance remained constant and similar to the control plants. In contrast to roots, 1 d of Pi re-supply did not cause a decrease in the abundance of any *AtPHT1* transcripts compared to Pi-deprived plants of the same age. Interestingly, the transcript abundance for *AtPHT1;8* and *AtPHT1;9* was actually higher after 1 d of Pi resupply than in the Pi-deprived plants of the same age. The transcript amount for most of the *AtPHT1* genes did eventually become repressed; however, the repression was not as strong as in roots, despite the fact that these plants had twice the shoot Pi concentration of the control plants (Additional file 1: Figure S1B).

**Figure 2:**
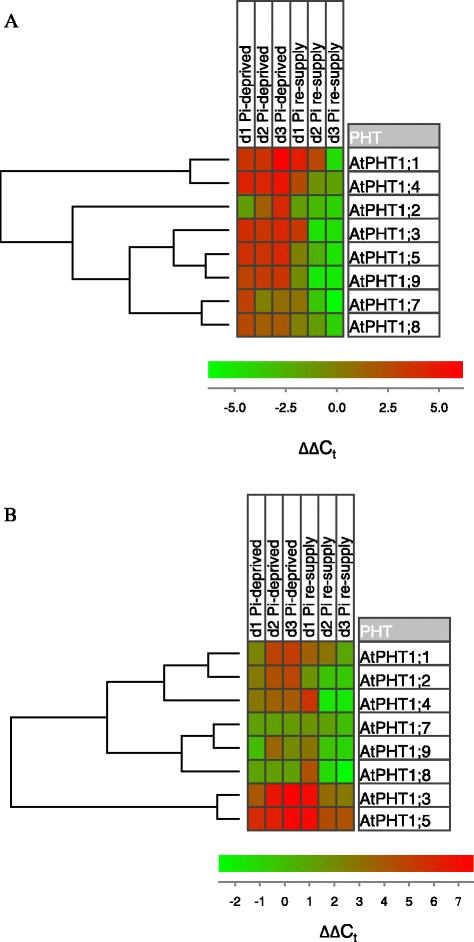
**Co-expression analysis of the** ***AtPHT1*** **gene family in response to Pi supply.** A hierarchical cluster analysis of *AtPHT1* gene transcript patterns in roots **(A)** and shoots **(B)** of the 42-day-old *Arabidopsis* plants described in the legend to Figure 1 is shown. ∆∆C_t_ values generated from the results shown in Figure 1 using plant organs continuously supplied with Pi as a reference were analysed using Euclidean distance and complete linkage [33].

Cluster analysis showed that the transcript responses to changes in Pi supply for the main *AtPHT1* transporter genes, *AtPHT1;1* and *AtPHT1;4*, along with *AtPHT1;2*, were distinct from those of the other *AtPHT1* genes in both roots and shoots (Figure 2). In roots, the response patterns for *AtPHT1;8* and *AtPHT1;7* were similar to each other, while the responses of *AtPHT1;9* were most similar to those of *AtPHT1;5* and *AtPHT1;3*. In the shoot, the response patterns for *AtPHT1;8* and *AtPHT1;9* clustered with those of *AtPHT1;7*, while the pattern of changes for *AtPHT1;3* and *AtPHT1;5* clustered discretely.

### Disruption of *AtPHT1;8* or *AtPHT1;9* had diverse effects in *Arabidopsis* seedlings

There is only a single mutant available for each *AtPHT1,8* and *AtPHT1;9* that has a predicted T-DNA insertion in the exon region. PCR across the predicted T-DNA left-border (LB) insertion sites and sequencing of the PCR products confirmed that the putative *Atpht1;1-2*, *Atpht1;8* and *Atpht1;9–1* mutants used in this study were homozygous for the presence of T-DNA at sites expected to disrupt gene function (Additional file 2: Figure S2A). At least two T-DNAs have been inserted in a head-to-head orientation in all three mutants. In *Atpht1;1–2*, two T-DNAs were inserted 42 bp downstream of the start of exon 3, confirming previous results (Additional file 2: Figure S2B) [26]. In the *Atpht1;8* mutant, the T-DNAs were located 595 bp downstream of the start of exon 2 (Additional file 2: Figure S2C). The insertion site in the previously characterised *Atpht1;9–1* mutant allele [28] was found to be located 77 bp upstream of the start codon in exon 1 (Additional file 2: Figure S2D). These insertions caused the amount of the corresponding transcript for each mutant gene to be severely reduced to below the limit of detection by semi-qPCR and just above the limit of detection by qPCR (Additional file 3: Figure S3).

**Figure 3:**
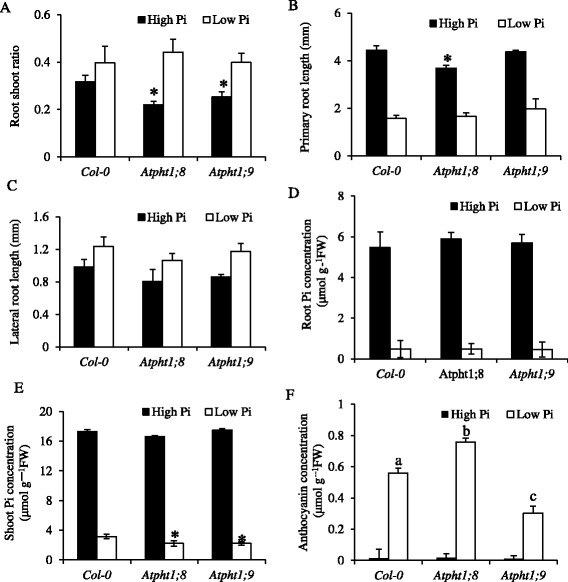
**Developmental and biochemical responses of** ***Arabidopsis*** **Col-0 and** ***Atpht1*** **knock-out seedlings to Pi limitation on solid medium.** Seedlings were grown for 5 d on solid medium containing 250 μM Pi before transfer to fresh medium containing either 250 μM Pi (High Pi) or 5 μM Pi (Low Pi) for 12 d. Root-to-shoot ratio **(A)**, primary root length **(B)**, lateral root length **(C)**, root Pi concentration **(D)**, shoot Pi concentration **(E)** and anthocyanin concentration **(F)** were determined at harvest. Values are means ± S.D. (n = 3 plates of 12 seedlings each). * or different letters indicate significantly different means (P <0.05) compared to the corresponding wild-type or each other according to two-way ANOVA and Tukey’s Multiple Comparison of Means, respectively.

The disruption of *AtPHT1;8* or *AtPHT1;9* gene function did not cause gross morphological changes in 17-day-old mutant seedlings supplied with sufficient Pi or depleted of Pi (Figure 3, Additional file 4: Figure S4). The most striking visible phenotype in both mutants supplied with sufficient Pi was a 20% to 30% reduction in root-to-shoot ratio compared to the corresponding Col-0 WT (Figure 3A). This was brought about by a 10% greater shoot biomass combined with a 10% lower root biomass in the mutants (Additional file 5: Figure S5). The decreased root biomass in the P-sufficient *Atpht1;8* mutant was at least partly due to a lower primary root length (Figure 3B), although the lateral roots also tended to be shorter in both mutants (Figure 3C). When depleted of P, the root system biomass of *Atpht1;8* and *Atpht1;9–1* seedlings was not significantly different from the WT (Figure 3, Additional file 5: Figure S5). The Pi concentration in the roots of the mutants was also the same as that in WT (Figure 3D), but it was 20% lower in the mutant compared to WT shoots (Figure 3E). The lower shoot Pi concentration might be expected to enhance P-starvation responses such as anthocyanin production which was indeed the case in the *Atpht1;8* mutant (Figure 3F). By contrast, the anthocyanin concentration in the shoots of *Atpht1;9–1* seedlings depleted of P was only about half of that in the WT. This lower anthocyanin concentration was not due to a dilution by growth, as shoot biomass in *Atpht1;9-1* seedlings depleted of P was about 10% less than that of WT seedlings (Additional file 5: Figure S5).

### Disruption of *AtPHT1;8* or *AtPHT1;9* compromised root-to-shoot translocation of Pi

**Figure 4:**
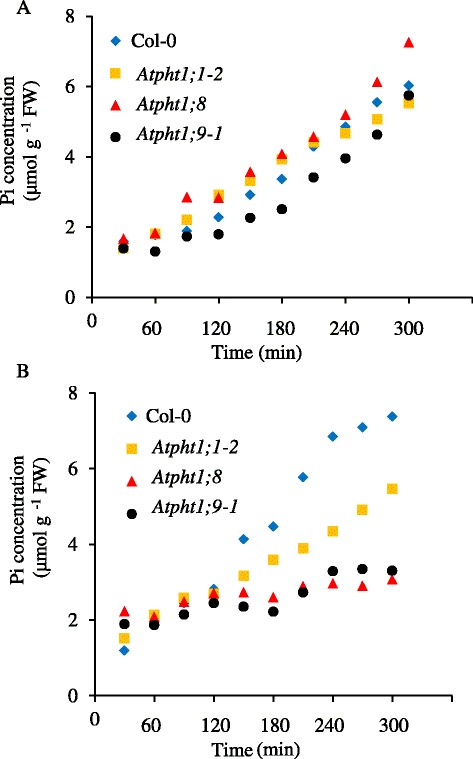
**Short-term accumulation of Pi by P-depleted 48-day-old Col-0 and** ***Atpht1*** **knock-out** ***Arabidopsis*** **plants grown in hydroponics.** Accumulation of Pi in roots **(A**) and shoots **(B)** of WT and *pht1* mutant plants over a 6-hour time course. Seedlings were grown in nutrient solution containing 250 μM Pi for 30 d, transferred to solution without Pi for 18 d to deplete plant P pools, and then transferred to solutions containing 250 μM Pi. Tissues were harvested every 30 mins for 6 h after the last transfer. Values are means ± S.D. (n = 3 biological replicates with 12 plants each grown at separate times). Error bars have been excluded for clarity, and can be viewed in Additional file 7: Figure S7.

Forty-eight-day-old *Atpht1;8* and *Atpht1;9–1* plants depleted of P were assessed for their short-term ability to remove Pi from the external nutrient solution in comparison to the WT and to the *Atpht1;1–2* mutant (Additional file 6: Figure S6). As expected, the *Atpht1;1–2* mutation caused a significantly lower rate of Pi removal from the nutrient solution than WT (Table 1). However, the *Atpht1;8* and *Atpht1;9–1* mutations had a negligible effect on the ability of P-depleted plants to remove Pi from the nutrient solution. Moreover, the *Atpht1;8* and *Atpht1;9-1* mutants were clearly distinguished from both WT and *Atpht1;1–2* in the short-term kinetics of Pi accumulation in the roots and shoots of these P-depleted plants upon Pi re-supply (Figure 4, Additional file 7: Figure S7). The *Atpht1;1–2* mutant, although compromised in its ability to acquire Pi from the nutrient solution (Table 1), had a root Pi concentrations similar to that of WT (Figure 4A). The concentration of Pi tended to be somewhat higher in the roots of *Atpht1;8* than in WT throughout the time course, while the Pi concentration in the roots of *Atpht1;9–1* tended to be marginally lower than in WT. After 300 min of Pi re-supply to the Pi-deprived plants, the roots of the *Atpht1;8* mutant had a significantly higher Pi concentration than those of WT, while the roots of *Atpht1;9–1* were indistinguishable from those of WT.

**Table 1:** **Capacity of 48-day-old P-depleted** ***Arabidopsis*** **WT and** ***Atpht1*** **knock-out plants to withdraw Pi from nutrient solution (see Additional file** 6 **: Figure S6)**

**Genotype**	**Rate of Pi removal** **(nmol Pi plant** ^**−1**^ **min** ^**−1**^ **) ± S.D.**	**R** ^**2**^
Col-0	99 ± 5	0.98
*Atpht1;1-2*	67 ± 5*	0.99
*Atpht1;8*	94 ± 12	0.96
*Atpht1;9-1*	92 ± 7	0.99

*Significantly different means (P <0.05) according to one-way.

ANOVA (n = 3 biological replicates with 12 plants each).

In sharp contrast to the roots, the shoots of all three P-depleted mutants accumulated dramatically less Pi than WT during short-term Pi re-supply (Figure 4B). The *Atpht1;1–2* mutant had noticeably lower shoot Pi concentrations than WT at all time points from 150 min onwards, as might be expected from the decreased ability of this mutant to remove Pi from the nutrient solution. However, both *Atpht1;8* and *Atpht1;9–1* plants, which were not compromised in acquiring Pi from the nutrient solution, accumulated much less of the added Pi in their shoots than either WT or the *Atpht1;1–2* mutant. After 300-min exposure to Pi, the shoot Pi concentration in both *Atpht1;8* and *Atpht1;9–1* mutants was less than half of that in WT and 40% lower than in the *Atpht1;1–2* mutant that was impaired in the primary uptake of Pi (Figure 4B).

**Figure 5:**
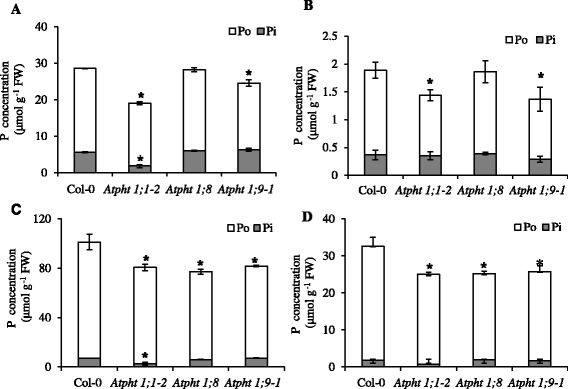
**The effect of Pi supply on the Po and Pi concentrations in 44-day-old** ***Arabidopsis*** **Col-0 WT and** ***Atpht1*** **plants grown in hydroponics.** The P concentrations in the inorganic (Pi) and organic (Po) pools were determined in roots **(A & B)** and shoots **(C & D)** of plants grown on nutrient solution containing a sufficient Pi supply **(A & C)** or no Pi supply **(B & D)**. Po was calculated as total P minus Pi. Plants were grown in nutrient solution containing 250 μM Pi for 30 d, and then transferred to solution containing 250 μM Pi or no added Pi for 14 d before harvest. For Po and Pi concentrations, values are means ± S.D. (n = 3 biological replicates of 12 plants each grown at separate times). *indicates significantly different means (P <0.05) relative to Col-0 according to one-way ANOVA followed by Tukey’s Multiple Comparison of Means.

The longer-term impact of the *Atpht1;1–2*, *Atpht1;8* and *Atpht1;9–1* mutations on plant P pools was determined in 30-day-old plants grown in the presence of Pi and then deprived or not of Pi for 14 d (Figure 5). For *Atpht1;1–2* plants with their compromised Pi uptake capacity (Table 1), the long-term total P concentration was lower than in WT in both roots and shoots of plants grown at either Pi supply. This lower total P concentration was due to lower concentrations of both Pi and the P esterified into acid-hydrolyzable organic compounds (Po). The Po was 35% lower in the root and 25% lower in the shoot compared to the WT regardless of the Pi supply, while the Pi concentration was only lower than WT in *Atpht1;1–2* roots and shoots under Pi-sufficient conditions. On the other hand, the long-term Pi concentration in the root and the shoot of the *Atpht1;8* mutant was indistinguishable from WT and *Atpht1;9–1* in both Pi-sufficient and Pi-limited conditions. The Pi concentration was also similar to *Atpht1;1–2* but in Pi-limited condition only. The total P concentration in roots of the *Atpht1;8* mutant was identical to the WT regardless of Pi supply, while in the shoots the total P concentration was lower and similar to that of the *Atpht1;1–2* mutant. This difference in root, but not shoot, total P concentration between these two mutants highlights a fundamental difference between them; the lower shoot P in *Atph1;8* compared to WT was not due to a lower P concentration in the root, as may have been the case in *Atpht1;1–2*, which is impaired in Pi uptake. In the roots of Pi-sufficient *Atpht1;9–1* plants, the Pi concentrations were similar to those in the *Atpht1;8* mutant, while the Po concentrations were similar to those in the *Atpht1;1–2* mutant. However, in the shoots of these plants, both Pi and Po concentrations were similar to those of *Atpht1;8*, indicating that *Atpht1;9* only differs from *Atpht1;8* in its ability to allocate Pi to Po in the roots. This difference was also observed in Pi-deficient plants (Figure 5B and D).

### Loss of *AtPHT1;8* and *AtPHT1;9* influenced the transcript profiles of other genes repressed by Pi

A panel of 17 Pi-responsive genes, including the full set of *AtPHT1* genes, was used to assess the interactions of *AtPHT1;8* and *AtPHT1;9* with other components of the P-starvation response (Additional file 8: Figures S8 and Additional file 9: Figure S9). The gene panel also included a sub-set of those genes reported to respond to either local or systemic signals generated by plant P status [18]. The genes in the panel that respond to distant systemic P signals were *AtPHT1;4, AtPHT1;5*, *AtPHT1;7*, *AtPHT1;8*, *AtPHO2, AtMIR399d, At4* and *AtIPS1*, while those that responded to local P status were *AtERF1*, *AtGSTU12*, *AtLOX4*, *At2-MMP* and *AtWRKY75*.

**Figure 6:**
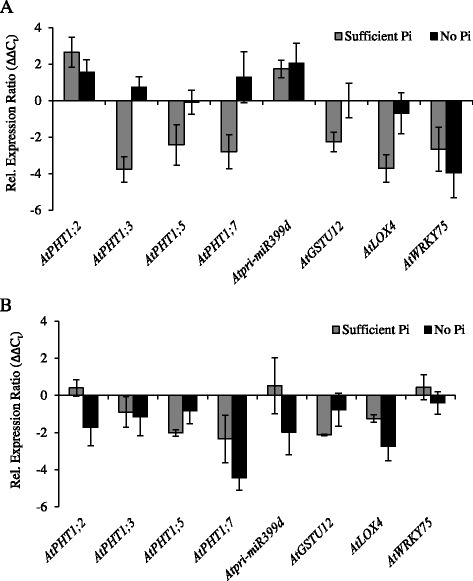
**Effect of the** ***Atpht1;8*** **mutation on the P-responsiveness of transcripts from Pi-responsive genes, including those encoding known P signaling components.** Transcript abundance in 44-day-old *Atpht1;8* plants is expressed relative to that in WT plants grown under the same conditions (∆∆C_t_ values). The transcript patterns were examined in both roots **(A)** and shoots **(B)**. Plants were grown in nutrient solution containing 250 μM Pi for 30 d, and then transferred to solution containing 250 μM Pi or no added Pi for 14 d before harvest. Each panel shows the comparison made using plants grown on nutrient solution containing sufficient Pi or no Pi. Values are means ± S.D. (n = 3 biological replicates of 12 plants each grown at separate times).

In 44-day-old plants, the *Atpht1;8* mutation caused changes in the transcript levels of some of the selected Pi-responsive genes (Figure 6, Additional file 8: Figure S8). In the roots of Pi-sufficient *Atpht1;8* plants, *AtPHT1;3, AtPHT1;5* and *AtPHT1;7* were more strongly repressed than in WT, while *AtPHT1;2* transcripts were more abundant (Figure 6A). However, transcripts from all the *AtPHT1* genes were induced to WT levels upon P depletion. *Atpri-MIR399d* transcripts were less strongly repressed in *Atpht1;8* roots at sufficient Pi than in WT roots, while transcripts from the set of genes influenced by local Pi signals were generally more strongly repressed consistent with higher Pi concentrations in these roots. P-depletion of the *Atpht1;8* mutant resulted in lower *AtWRKY75* transcript levels in the roots compared to WT. However, the abundance of these transcripts did not respond to changes in Pi supply in the roots of the mutant. Thus, *AtWRKY75* transcript levels were also lower in this tissue than in the WT in the presence of Pi.

The shoots of *Atpht1;8* plants grown in the presence of sufficient Pi contained less *AtPHT1;5* and *AtPHT1;7* transcripts than WT (Figure 6B). In P-depleted plants, the abundance of *AtPHT1* transcripts, including those from *AtPHT1;5*, were generally de-repressed to the same levels seen in the WT. The exception was transcripts from *AtPHT1;7,* which were not as strongly de-repressed as in WT. In *Atpht1;8* shoots, the only observed change in transcript profile for genes responsive to systemic signals of P status was a slight decrease in the strength of the de-repression of *Atpri-MIR399d* transcripts in the P-depleted plants. Among the genes responsive to local P signals, *AtGSTU12* was somewhat more repressed in Pi-sufficient *Atpht1;8* shoots compared to WT, but this repression was overcome during P-depletion. By contrast, *AtLOX4* transcripts were not as strongly de-repressed by P depletion in the mutant as in the WT.

**Figure 7:**
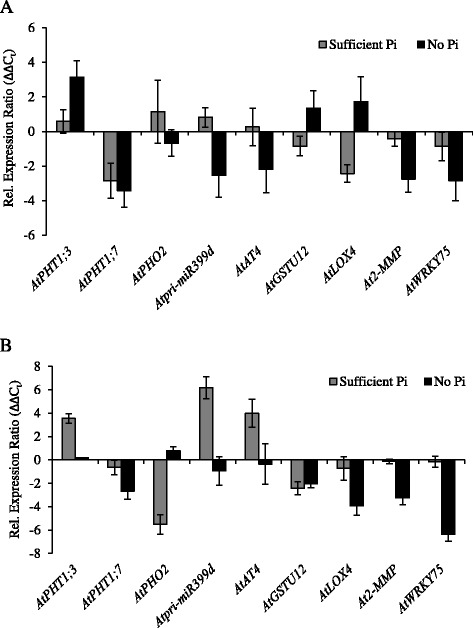
**Effect of the** ***Atpht1;9–1*** **mutation on the P-responsiveness of transcripts from Pi-responsive genes, including those encoding known P signaling components.** Transcript abundance in 44-day-old *Atpht1;9–1* plants is expressed relative to that in WT plants grown under the same conditions (∆∆C_t_ values). The transcript patterns were examined in both roots **(A)** and shoots **(B)**. Plants were grown in nutrient solution containing 250 μM Pi for 30 d, and then transferred to solution containing 250 μM Pi or no added Pi for 14 d before harvest. Each panel shows the comparison made using plants grown on nutrient solution containing sufficient Pi or no Pi. Values are means ± S.D. (n = 3 biological replicates of 12 plants each grown at separate times).

The accumulation of transcripts from the target genes in 44-day-old *Atpht1;9–1* plants was similar to, but distinct from, that in the *Atpht1;8* mutant (Figure 7, Additional file 9: Figure S9). In the roots of Pi-sufficient *Atpht1;9–1*, *AtPHT1;7* and *AtLOX4* transcripts were less abundant than in WT (Figure 7A). During P depletion, *AtPHT1;7* was less strongly de-repressed than in WT, while *AtPHT1;3* transcripts were more abundant. *Atpri-MIR399d* and *AtAT4* transcripts were less abundant in the P-deprived *Atpht1;9–1* mutant. Interestingly, both *At2-MMP* and *AtWRKY75* transcript amounts were lower in response to P depletion in the *Atpht1;9–1* mutant, similar to what was observed for *AtWRKY75* in the roots of the *Atpht1;8* mutant.

In the shoot of P-sufficient *Atpht1;9–1*, *AtPHT1;3* was less repressed than in WT (Figure 7B). Transcripts from *AtPHO2* were more strongly repressed by Pi in the mutant than in WT which was complemented by a decreased repression by Pi for both *Atpri-MIR399d* and *AtAT4* transcripts. In the shoot of P-depleted *Atpht1;9–1*, *AtPHT1;7* transcripts, along with those from the entire set of genes associated with local signaling events, were less de-repressed than those in the WT.

## Discussion

**Figure 8:**
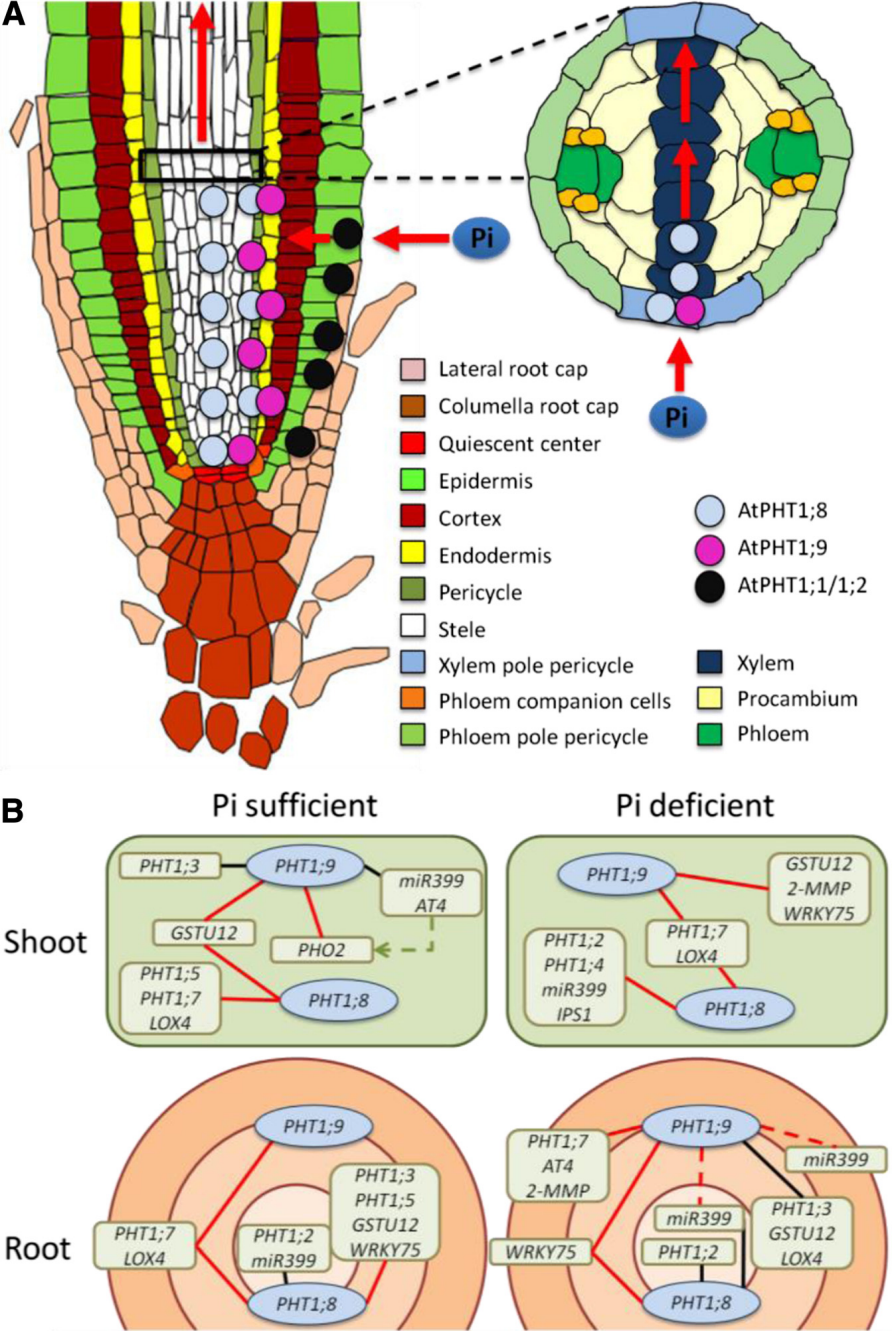
**Proposed location in the root and genetic interactions of AtPHT1;8 and AtPHT1;9. (A)** Proposed root-to-shoot translocation pathway for Pi through the sequential action of AtPHT1;9 and AtPHT1;8. The endodermis and/or pericycle are the most likely locations for AtPHT1;9. AtPHT1;8 appears to act in cell layers deeper in the root than AtPHT1;9, perhaps in the pericycle or xylem. Two potential translocation pathways are envisaged. The location of AtPHT1;9 and/or AtPHT1;8 in the xylem pole pericycle would facilitate the transfer of Pi into the xylem for translocation to the shoot. (Image adapted from http://www.ens-lyon.fr/RDP/SiCE/Resources.html). **(B)** Summary of the observed genetic interactions of AtPHT1;8 and AtPHT1;9 with other Pi-responsive genes. The transcripts from the indicated genes were either more abundant (black lines) or less abundant (red lines) in the respective PHT1 knock-out lines than in the wild type. The genetic interactions are superimposed onto the relative position proposed for AtPHT1;8 and AtPHT1;9 in the root. The diagram takes into consideration that the P concentration in each mutant is likely to be higher on the exterior side than on the interior side of the missing protein than in the wild-type. For example, since all the genes tested for interactions were repressed by Pi supply, gene transcripts that were expressed at a lower level in a mutant were positioned toward the exterior of the root, where the P concentration was presumed to be higher in the mutant than in the wild type. Such an assignment could not be made for pri-miR399d (red dotted lines) in roots of Pi-deprived seedlings, indicating our imperfect understanding of the regulation of pri-miR399 expression. The repression of AtPHO2 in leaves of Pi-sufficient Atpht1;9-1 may be in response to changes in the expression of the regulatory RNAs mir399d and AT4 (green dotted arrow).

A clear response in *Arabidopsis* to changes in P status is the reversible repression of *AtPHT1* gene expression [20]. The modulation of *AtPHT1* expression alters the Pi transport activity within the plants in response to the prevailing Pi availability [10]. Here we provide evidence that *AtPHT1;8* and *AtPHT1;9* were instrumental in the movement of Pi from the root to the shoot. In addition, we show that these genes had overlapping but distinct functions, as well as interactions at the transcript level with *AtPHT1;7* and other genes that are involved in controlling P nutrition (Figure 8).

### *AtPHT1;8* and *AtPHT1;9* are necessary for the root-to-shoot translocation of Pi

Previous work indicated that both AtPHT1;8 and AtPHT1;9 are involved in Pi uptake from the external medium, but that neither gene has a role in translocating Pi to the shoot [28]. This conclusion was based on the observation that the shoots of mutant plants supplied with adequate Pi had the same Pi concentration as wild-type plants. By contrast, plants that were supplied with inadequate Pi had genotype-dependent differences in shoot Pi concentration. These differences in shoot Pi accumulation were concluded to be a direct consequence of the Pi concentration in the roots governing the Pi concentration in the leaves. However, as discussed below, our short-term Pi uptake experiments show that mutations in either *AtPHT1;8* or *AtPHT1;9* severely compromised the ability of *Arabidopsis* to translocate Pi to the shoot, explaining the lower total P concentrations that we also observe in the shoots of these mutants compared to WT.

As expected, our results showed that the rate of Pi uptake from solution is markedly reduced in the *Atpht1;1–2* mutant compared to the WT. Since *AtPHT1;1* is one of the main paths for Pi entry into the root during low Pi availability [26], it is not surprising that the lower uptake capacity in this mutant would compromise the amount of Pi translocated to the shoot in the short term. The lower Pi concentrations observed in the shoots in the short term are likely to be due to the roots of P-starved seedlings retaining a higher proportion of the available Pi to satisfy root growth at the expense of Pi translocation to support shoot growth. On the other hand, the lower shoot Pi and Po observed in the longer term in the shoots of this mutant are probably a direct consequence of the decreased P concentrations observed in the roots.

In contrast to *Atpht1;1–2*, both *Atpht1;8* and *Atpht1;9–1* mutants had the same Pi-uptake rate as WT, which is also not surprising since PHT1;1 would be fully functional in both mutants. However, the short-term accumulation of Pi in the shoots of *Atpht1;8* and *Atpht1;9–1* was much slower than in the *Atpht1;1–2* mutant, suggesting a role in root-to-shoot translocation. This interpretation is supported by the following. During longer-term growth under P sufficient conditions, roots of *Atpht1;8* had WT levels of both Pi and Po, while Po was only slightly compromised in roots of *Atpht1;9–1*. Therefore, the roots of *Atpht1;8* were just as capable of extracting Pi and allocating it to Po as those of WT and the roots of *Atpht1;9–1* nearly so. On the other hand, Po, but not Pi, concentrations were compromised in the shoots of the *Atpht1;8* and *Atpht1;9–1* plants, whereas the concentrations of both these P pools were compromised in the shoots of *Atpht1;1–2*. Together, these observations demonstrate that *AtPHT1;8* has little, if any, role in the uptake of Pi, but instead is necessary for the root-to-shoot translocation of Pi. The same is probably true for *AtPHT1;9*. However *Atpht1;9-1* has a somewhat lower total P concentration in the root than WT, which is due to a compromised ability to allocate Pi to root Po. While this could be due to decreased Pi uptake or the reduced export of Po from the shoot, it is much more likely given our results to be due to AtPHT1;9 acting more prominently in an outer cell layer of the central cylinder of the root relative to AtPHT1;8 (Figure 8). This situation would result in a lower Pi flux into the interior of the root for *Atpht1;9–1* than for *Atpht1;8* and a change in the balance of Pi allocation that favours translocation to the shoot over the formation of Po in the root. Thus, AtPHT1;8 and AtPHT1;9 may operate analogous to the rice OsPht1;2 transporter, which also functions mainly in the translocation of Pi from the root to the shoot [34]. However, the two *Arabidopsis* proteins act sequentially, with AtPHT1;9 acting in cell layers closer toward the root surface than AtPHT1;8 (Figure 8).

Differences in the expression profiles of *AtPHT1* genes that could lead to differences in function are apparent in a spatiotemporal gene expression map of the *Arabidopsis* root [35], where the resolution of the original microarray-based map has been substantially increased by additional microarray datasets (see Brady et al. [36] and references therein). The plants used to generate the expression map were grown on solid medium containing 1.25 mM Pi. Therefore, their tissues would have been saturated with Pi, strongly suppressing *PHT1* gene expression. These seedlings, therefore, contrast with the Pi-deprived seedlings with high *PHT1* expression that we used to discover that the movement of Pi from the root to the shoot is controlled by *AtPHT1;8* and *AtPHT1;9*.

Visualising the root gene expression map using the *Arabidopsis* eFP browser [37], *PHT1* transcript abundance in seedlings grown under high Pi conditions is especially low in the meristematic region of the root, from the columellar root cap nearly to the elongation zone (columella + sections 1 to 5 in Brady et al. [36], Additional file 10: Table S1). This is in contrast to the region extending from the top-most section of the meristematic region through the elongation zone into the maturation zone (sections 6 to 12 in Brady et al. [36], Additional file 10: Table S1). The low meristematic expression of *PHT1* transcripts holds for all cell layers except the procambium. In the meristematic zone, *AtPHT1;8* and *AtPHT1;9* transcripts are generally as abundant, if not more abundant, than the transcripts recognised by the redundant microarray probe sets that detect the sum of *AtPHT1;1* + *AtPHT1;2* transcripts or the sum of *AtPHT1;4* + *AtPHT1;7* transcripts (Additional file 10: Table S1). This is particularly the case in the endodermal cell layer surrounding the stele [35,36] (Additional file 10: Table S1). This transcript pattern suggests the endodermis of the meristematic region as a potential gateway where the control of Pi transport could be mediated by *AtPHT1;8* and/or *AtPHT1;9* upon their de-repression at low Pi availability (Figure 8A).

The *PHT1* expression patterns within the cells of the stele suggest other potential sites where *AtPHT1;8* and *AtPHT1;9* may regulate Pi transfer into the xylem (Figure 8A). Within the stele as a whole, transcripts from *AtPHT1;8* and *AtPHT1;9* do not predominate [35]. However, the higher-resolution gene expression data provided by Brady et al. [36] at the cellular level shows that this is largely due to a dilution effect caused by relatively high levels of transcripts from several other *PHT1*genes in the procambium (Additional file 10: Table S1). *AtPHT1;8* and/or *AtPHT1;9* transcripts are more abundant relative to those from other *AtPHT1* genes within the xylem of the meristematic region, at least in these Pi-satiated plants. A similar high relative abundance of *AtPHT1;8* and/or *AtPHT1;9* transcripts also exists in the xylem pole pericycle cell strand. In this case, the high relative abundance of *AtPHT1;8* and/or *AtPHT1;9* transcripts extends from the root tip, through the meristematic and elongation zones into the maturation zone. This expression pattern, then, offers the potential for the xylem pole pericycle cell strand to regulate Pi transport along a large section of the root (Figure 8A). *AtPHT1;8* and/or *AtPHT1;9* transcripts are also relatively abundant compared to those from other *PHT1* genes in the protophloem + metaphloem and in the phloem companion cells within the meristematic region. If *AtPHT1;8* and/or *AtPHT1;9* are involved in controlling Pi transport in these cells, it is likely in response to the flow of Pi back to the root tip from the aerial tissues through the phloem, rather than to the flow from the root to the shoot through the xylem (Figure 8A).

From the available results, it seems likely that the cells of the endodermis, the xylem pole pericycle, or the xylem itself, are where AtPHT1;8 and AtPHT1;9 could provide a barrier to Pi transport. This barrier would separate the external solution from the vasculature with AtPHT1;9 acting in a cell layer closer to the root surface than AtPHT1;8 (Figure 8A). Confirmation of the cell types in which AtPHT1;8 and AtPHT1;9 regulate the flow of Pi to the shoot awaits a high-resolution gene expression map of the root from seedlings in which *PHT1* gene expression is highly de-repressed by Pi deprivation. However, a full picture of how AtPHT1;8 and AtPHT1;9 regulate the flow of Pi to the shoot will require a full understanding of the biochemistry of these transporters, including any regulation of their activity by post-translational modification or by interactions with other proteins, including other PHT1 proteins [10].

### Absence of AtPHT1;9 modulates the activity of the PHO2 regulon

Our results support and extend the proposed interactions of both *AtPHT1;8* and *AtPHT1;9* with the PHO2 regulatory circuit [14,22]. *AtPHO2* encodes an ubiquitin conjugating enzyme implicated in repressing Pi uptake when Pi availability is high [14,21,22]. Its expression is in turn repressed by the phloem-mobile microRNA Atmir399 [38], a component of the systemic P-signaling system. Atmir399 is synthesised in the shoot and translocated to the roots when shoot P status is low [39,40]. Atmir399 functions by binding to and providing a substrate for the specific cleavage and degradation of *PHO2* transcripts. The activity of Atmir399 is itself modulated by regulatory RNAs *AtIPS1* and *AtAT4*, which are thought to mimic the *AtPHO2* transcript and compete for Atmir399 binding [23]. Together, AtPHO2 and these small RNAs monitor the plant P status [40-42]. However, the interactions of the *AtPHO2*, *AtMIR399, AtIPS1* and *AtAT4* gene products are complex, given that the expression of the *AtMIR399* gene family as well as *AtIPS1* and *AtAT4* are all de-repressed by low Pi availability [14,22,24]. A decrease in AtPHO2 activity, either by mutating or suppressing *AtPHO2*, or by ectopic over-expression of *AtMIR399*, causes Pi to accumulate in the shoots [14,22,43,44]. Interestingly, both *AtPHT1;8* and *AtPHT1;9* are up-regulated in the *Atpho2* mutant and in *AtMIR399* over-expression lines [14,22]. Therefore, our results indicating that AtPHT1;8 and AtPHT1;9 are necessary for root-to-shoot Pi translocation are consistent with these Pi transport proteins being directly involved in the increased translocation of Pi to the shoots in the transgenics with decreased *AtPHO2* expression.

We found that the amounts of *Atpri-MIR399d* and *AtAT4* transcripts in the shoots of *Atpht1;9–1* were not as strongly suppressed by Pi as in the WT which may in turn explain the decrease in the amount of *AtPHO2* transcript in these shoots (Figure 8B). The abundance of transcripts from these three regulatory genes was consistent with the lower organic shoot P status of *Atpht1;9–1*. Interestingly, the Pi concentrations in the roots and shoots of these plants were the same as those in WT. Therefore, the modulation of these components of the AtPHO2 regulatory circuit were either being influenced by highly localized Pi concentrations, by an organic P ester, or by the absence of the AtPHT1;9 protein itself. The *Atpht1;8* mutation had little impact on the PHO2 regulatory circuit other than a modest modulation in the amounts of the *AtMIR399d* primary transcript, further differentiating the activity of AtPHT1;8 and AtPHT1;9.

### AtPHT1;8 and AtPHT1;9 activities govern the expression of *AtPHT1* genes involved in inter-organ Pi transport

Transcript analysis in the *Atpht1;8* and *Atpht1;9–1* mutant backgrounds revealed numerous genetic interactions between these and the other *AtPHT1* genes that are repressed by Pi (Figure 8B). These findings contradict an earlier report where *AtPHT1;8* and *AtPHT1;9* did not genetically interact with other Pi-starvation induced genes in plants of low P status [28]. However, that earlier study used end-point PCR to quantify transcripts which is a less sensitive method of transcript quantification than real-time PCR. Moreover, the transcript analysis was done on whole seedlings, while in our study roots and shoots were analysed separately and at two Pi supply levels. Together, these methodological differences in the earlier study may have masked the differences in transcript profiles that we report here.

The interactions of *AtPHT1;8* and *AtPHT1;9* with other genes were overlapping (Figure 8B), with 75% identity in the set of genes that showed significant changes in gene expression in both mutant lines compared to WT. The differences in effect of these two genes on other genes is a further indication that *AtPHT1;8* and *AtPHT1;9* are functionally distinct, consistent with the view that they act sequentially in Pi translocation. The interactions were apparently independent of organ Pi levels, since the long-term Pi concentrations in the *Atpht1;8* and *Atpht1;9–1* mutants were nearly indistinguishable from those in the WT. The interactions did, however, correlate with lower organ Po concentrations, indicating that the plants were not as highly P satiated as WT plants. Thus, a signal other than organ-level Pi concentration was likely to be responsible for the observed co-expression patterns.

The interactions of *AtPHT1;8* and *AtPHT1;9* with other *AtPHT1* genes excluded *AtPHT1;1*, *AtPHT1;2* and *AtPHT1;4*, which encode transporters that are important for Pi uptake from the soil solution [12,26]. Most of the interactions were instead with *AtPHT1;3*, *AtPHT1;5* and *AtPHT1;7*(Figure 8B), suggesting that the encoded transporters, like AtPHT1;8 and AtPHT1;9 themselves, are likely important for inter-organ Pi transport processes. This role has already been demonstrated for AtPHT1;5 [27]. Interestingly, *AtPHT1;3*, *AtPHT1;5*, *AtPHT1;7*, *AtPHT1;8* and *AtPHT1;9* were also among a very small group of PHR1-regulated genes that displayed a very strong and specific response to Pi availability in the root environment, with only AtPHT1;7 also responding equally well in shoots, as shown previously [32]. There was no evidence for a genetic interaction between *AtPHT1;8* and *AtPHT1;*9 themselves in our study. This is interesting, given that the two transporters appear to act sequentially in the transport of Pi to the shoot.

The lack of a strong alteration in transcript abundance for other *AtPHT1* genes in the roots of the *Atpht1;8* mutant under Pi deficiency was a further indication that the disruption of AtPHT1;8 function was directly responsible for the lower capacity for Pi translocation to the shoot in this mutant. By contrast, the alterations in *AtPHT1;3* and *AtPHT1;7* transcript abundance in the roots of *Atpht1;9–1* under Pi deficiency leaves open the possibility that the genetic interaction of *AtPHT1;9* with these genes is necessary for Pi translocation in WT. The stronger repression compared to that in WT of *AtPHT1;3*, *AtPHT1;5* and *AtPHT1;7* in *Atpht1;8* roots and of *AtPHT1;7* in *Atpht1;9–1* roots under adequate Pi supply indicated that decreases in the amounts of these other AtPHT1 proteins may also have contributed to the lower P accumulation in mutant shoots. A better understanding of the precise location of the various PHT1 proteins within the plant is necessary to more thoroughly interpret these findings.

The genes involved in local Pi signaling were even more strongly repressed by high Pi in the *Atpht1;8* mutant than in the WT (Figure 8B). The stronger repression in roots compared with that in shoots hinted at a possible accumulation of Pi or down-stream metabolites in roots due to reduced Pi transport into the shoot. The observed repression of *AtWRKY75* in low-Pi roots and *AtLOX4* in shoots of the same treatment could indicate that the underlying “local” P regulatory circuits trigger different downstream responses in different organs. For example, *AtLOX4* repression may lead to reduced shoot growth in low-Pi conditions, given that the *AtLOX4* gene is involved in the biosynthesis of jasmonic acid, an effector of shoot growth [45].

### Integration of PHT1 gene expression and tissue Pi status

The transcriptional responsiveness of *AtPHT1;8* and *AtPHT1;9* to changes in Pi supply was similar to each other, but not identical. Feeding Pi to Pi-deprived plants suppressed *AtPHT1;9* transcripts in the roots more quickly and more strongly than *AtPHT1;8* transcripts. By contrast, the suppression of *AtPHT1;8* transcripts in the shoots of these same plants was stronger than that for *AtPHT1;9*, although the overall response of P-responsive transcripts in the shoots was much weaker than that in the roots.

For both *AtPHT1;8* and *AtPHT1;9*, and indeed nearly all of the *AtPHT1* genes, the strong repression in the roots upon Pi re-supply brought the transcript levels far below those found in control plants continually fed Pi. This would be a useful adaptation for plants in their natural environment, where the available Pi concentration in the soil rarely exceeds 10 μM [1,46]. Plants growing under these soil conditions would most certainly not be Pi-limited, but there would be strong *PHT1* expression and a danger of Pi over-accumulation if a rich supply of Pi was encountered. Thus, should a Pi-limited plant encounter a very Pi-rich soil patch, the rapid and strong systemic suppression of *AtPHT1;9* in the roots followed by the suppression of *AtPHT1;8* and the other *AtPHT1* genes would prevent Pi toxicity through the rapid decrease in the influx of Pi into the shoots. The observed stronger suppression of all *AtPHT1* transcripts in the roots upon Pi re-supply could be due to a systemic signal, given that the nutrient solution is quickly depleted of Pi and Pi accumulation is much stronger in the shoot than in the root itself (Jost *et al*., personal communication). By contrast, the weaker over-all suppression of *AtPHT1* transcript abundance in the shoots could be a response to the lower Pi accumulation in the root, more slowly increasing Po concentrations or to the lower local accumulation of Pi in the cytosol of shoot cells, given that most of the Pi translocated to the shoot would immediately be moved to the vacuole [47]. The observed increase in *PHT1* transcript abundance in the shoot after 1 d of Pi re-supply was consistent with this proposed delay in Pi signal perception in the shoot.

### Loss of *AtPHT1;8* and *AtPHT1;9* did not intensify the morpho-physiological response to Pi limitation

Plants respond to changes in Pi supply by altering their physiology and biochemistry, both of which are modulated by changes in gene expression [48,49]. Key physiological characteristics associated with low Pi availability are a relative increase in resource allocation to the roots resulting in an increased root-to-shoot ratio as well as increased root-hair proliferation, root branching and lateral root elongation [50-52]. The deployment of these traits is often accompanied by an arrest in primary root elongation. In our system, inactivation of *AtPHT1;8* and *AtPHT1;9* had little effect on the establishment of these traits.

Our conclusions regarding the morpho-physiological responses of the *Atpht1;8* and *Atpht1;9–1* mutants to changes in Pi status was somewhat contrary to a previous report on these two genes [28]. In the earlier report, *Atpht1;9* mutants, including the *Atpht1;9–1* knock-out allele examined here, and *AtPHT1;9* / *AtPHT1;8* RNAi co-suppressed lines, showed increased P-starvation responses compared to those in WT. These enhanced responses included effects on primary root length, lateral root length and shoot FW [28]. The disparity between our studies could be due to differences in the plant growth conditions. For example, Remy et al. [28] used a medium containing 100 μM Fe, while our medium contained 40 μM Fe. High Fe concentrations can lead to the precipitation of Pi thus aggravating P limitation responses. Previous work showed that the inhibition of primary root growth during Pi limitation of *Arabidopsis* grown on synthetic media was due to Fe toxicity and that the recovery from inhibition was more rapid for larger plants [53]. Thus, our larger plants seem to have been quite immune to the Fe exposure in the medium used here. Other differences in growth conditions that may have led to study-dependent differences in plant root architecture under Pi limitation were the gelling agent [54] and the presence or absence of myo-inositol, a precursor for many secondary messenger molecules and a regulator of plant growth responses [30,55].

## Conclusion

The products of the *AtPHT1;8* and *AtPHT1;9* genes are involved in the translocation of Pi from the roots to the shoot in *Arabidopsis*. When supplied with moderate Pi for an extended period, the reduced Pi translocation to the shoots in *Atpht1;8* and *Atpht1;9–1* did not affect shoot Pi concentration, but instead lowered the organic P concentration. Some of the observed effects on growth and P allocation in the *Atpht1;8* and *Atpht1;9–1* mutants may be indirect, as mutations in these genes caused changes to the transcript abundance of other *AtPHT1* genes. The loss of AtPHT1;9 also had an indirect effect on the transcript abundance of genes involved in systemic signalling mediated by the PHO2/mir399 network. Thus, AtPHT1;8 and AtPHT1;9 have a wider role in the control of P nutrition in *Arabidopsis* than was previously reported.

## Methods

### Plant material and growth

*Arabidopsis* ecotypes Col-0 and T-DNA insertion lines SALK 088586C (*Atpht1;1–2*, At5g43350), SALK 056529 (*Atpht1;8*, At1g20860) and SALK 050730 (*Atpht1;9–1*, At1g7643) were obtained from the *Arabidopsis* Biological Resource Centre. Seeds were surface-sterilized in 70% (v/v) ethanol for 2 mins and 5% (v/v) sodium hypochlorite for 5 mins followed by five washes in sterile water for 5 mins each. Seeds were stratified at 4°C in the dark for 16 h before sowing. When determining the location of the T-DNA insertion, seeds were sown on solid B5 medium (Phytotechnology Laboratories, Kilsyth, VIC, Australia) containing 3% (w/v) sucrose. Seedlings were grown with the plates held in a vertical position.

For hydroponic growth of plants, a modified nutrient solution approximating one-half strength Hoagland’s solution [56,57] was used. The solution contained 2 mM Ca(NO_3_), 2 mM KNO_3_, 0.5 mM NH_4_NO_3_, 0.5 mM MgSO_4_, 0.25 mM KH_2_PO_4_, 50 μM KCl, 40 μM of Fe-EDTA, 25 μM of H_3_BO_3_, 2 μM MnCl_2_, 2 μM ZnSO_4_, 0.5 μM CuSO_4_, 0.15 μM of CoCl_2_ and 0.075 μM NH_4_Mo_7_O_24_. The seeds were sown on rock wool as described [58].

For the initial Pi re-supply experiments, Col-0 plants were grown in hydroponics. In the first four weeks, the nutrient solution was exchanged three times. In the depletion phase, nutrient solutions were exchanged twice a week for both the control and experimental plants. The controls were on nutrient solution containing 250 μM Pi while the experimental plants were on nutrient solution lacking Pi. After 12 days (d) of Pi deprivation on nutrient solution without Pi, experimental plants were transferred either to solutions containing 250 μM Pi or to solutions lacking Pi. To ensure a stable osmotic environment the solutions were exchanged daily during this period.

For the long-term Pi depletion study, the standard nutrient solution was changed once within the first four weeks of hydroponic growth, then alternating on every 2 d or 3 d for plants grown on Pi-containing nutrient solution, or every 7 d for plants grown on nutrient solution lacking in Pi. Tissues were harvested after 14 d of treatment.

For the experiment to measure the short-term rate of Pi depletion from the medium the seedlings were grown in nutrient solution containing 250 μM Pi, exchanged three times within 30 d. The seedlings were then transferred to solutions without Pi to deplete plant P pools, where the nutrient solution was changed every 7 d. After 18 d of Pi deprivation plants were then transferred to solutions containing 250 μM Pi. Tissues were harvested every 30 mins for 6 h after the last transfer.

For root-growth assays plants were grown on vertical plates of solid one-half-strength Hoagland’s nutrient solution containing 0.5% sucrose and 8 g/L of agar (Plant Micro Agar, Phyto Technologies Laboratory LLC, Shawnee Mission, Kansas, USA). Seedlings were grown for 5 d on solid medium containing 250 μM Pi before transfer to fresh medium containing either 250 μM Pi (High Pi) or 5 μM Pi (Low Pi) for 12 d.

All plants were grown with 10 h light (160 μmol m^−2^ s^−1^ PAR) at 21°C and 14 h dark at 18°C. Plant tissues were harvested with appropriate staggering starting 3 h after the beginning of the light period to ensure that plants were at a comparable physiological and metabolic state for each harvest.

For seed production, seedlings were transferred to pots with soil after 3 weeks and grown for a month under 10 h/14 h short-day conditions as above, before transfer to long-day conditions (16 h light [100 μmol m^−2^ s^−1^ PAR] at 22°C and 8 h dark at 19°C) until harvest.

### Characterization of T-DNA insertion mutant lines

Genomic DNA was isolated from leaves of 4-week-old plants using a cetyl trimethylammonium bromide (CTAB)-based method [59]. PCR genotyping was performed using primers specific for the T-DNA left border and gene-specific primers corresponding to the regions of At5g43350 (*AtPHT1;1*), At1g20860 (*AtPHT1;8*) and At1g76430 (*AtPHT1;9*) reported to be flanking the T-DNA inserts (see Additional file 11: Table S2).

### RNA isolation and quantitative reverse-transcription PCR

Poly-A^+^ RNA was isolated from up to 40 mg of plant tissue using oligo-dT magnetic beads (Dynabeads, Invitrogen Life Technologies, Grand Island, NY), followed by cDNA synthesis (Bioscript RT, Bioline, Alexandria, NSW) on the beads [60]. Primers for quantitative reverse-transcription PCR (qRT-PCR) (see Additional file 11: Table S2) were designed against candidate cDNA sequences using two software packages (Primer Express, Applied Biosystems, Life Technologies; BLAST Primer3, http://www.ncbi.nlm.nih.gov/tools/primer-blast). PCR primer efficiencies were determined using LinRegPCR [61]. Efficiencies did not change significantly between different cDNA samples (Additional file 11: Table S2). *AtPDF2* [29], *AtYLS8* [30] and *AtACT7* (At5g09810) were used as reference genes. qRT-PCR was carried out using a commercial reagent kit according to the manufacturer’s instructions (Power SYBR Green Master Mix, Applied Biosystems, Life Technologies). Reactions (10 μl) were performed in 96-well format and contained approx. 0.5 ng cDNA, 2.5 μl of a mixture containing 1.2 μM each of the forward and reverse primers, and 5 μl of master mix. The PCR conditions were one cycle of 50°C for 20 sec and 95°C for 10 min, followed by 40 cycles of 95°C for 15 sec and 60°C for 1 min (7500FAST Sequence Detection System, Applied Biosystems, Life Technologies) followed by a melt curve analysis to detect non-specific amplification products. The cycle threshold (C_t_) and normalized fluorescence values were determined for each sample during the quantitative PCR cycling reaction (Prism Sequence Detector Software v. 2.0, Applied Biosystems, Life Technologies). The threshold fluorescence value for determining C_t_ was set to 0.2 relative units. A comparative C_t_ approach was applied, where the transcript level of a target gene was normalized to the average signal from the *AtPDF2*, *AtACT7* and *AtYLS8* reference genes [29]. Expression levels were given on a log_2_ scale expressed as 40-∆C_t,_ where ∆C_t_ is the difference between the C_t_ of the target gene and the average C_t_ reference genes [22]. Therefore, 40-∆C_t_ value equal to 40 represents a transcript amount that is equal to the average transcript abundance for the reference genes. The threshold 40-∆C_t_ value in this experiment was 25, using a theoretical lowest target C_t_ value of 40. According to the manufacturer’s instructions, a C_t_ value of 35 (corresponding to a 40-∆C_t_ value of 30) marks the lower detection limit of the PCR instrument used. Changes in transcript levels compared to the corresponding tissues from plants continuously supplied with Pi are given on a log scale expressed as ∆∆Ct [62,63].

### P determinations and elemental analysis

Pi was measured using a modified ammonium molybdate method [64]. Fresh tissues were homogenized at a ratio of 1 mg sample fresh weight to 10 μl 1% (v/v) acetic acid. The homogenate was centrifuged twice at 12,000 × g for 15 min at 4°C. Samples were diluted and a 90 μl aliquot was combined with 210 μl 0.35% (w/v) NH_4_MoO_4_, 1.4% (w/v) ascorbic acid in 1 N H_2_SO_4_ and incubated in the dark for 60 min at 37°C. A standard curve was constructed using dilutions of KH_2_PO_4_. The absorbance of the reaction products was measured at 820 nm and the Pi concentration of the unknown sample extrapolated from the standard curve.

Total P was extracted from approx. 10 to 20 mg of dried tissue by digesting in 3 ml HNO_3_ at 100°C for 10 min. After cooling for 5 min, 1 ml of HClO_4_ was added. The acid digests were heated to 150°C until the vigorous reaction that takes place between the HClO_4_ and the organic residue had run to completion. Samples were heated to 180°C for 10 min to dehydrate any silica present. After the sample had cooled, 2 ml of de-ionized water were added. Samples were diluted as necessary before combining 150 μl with 50 μl malachite green and polyvinyl alcohol mixture [65] and allowed to stand for 8 min. The absorbance of the reaction was measured at 650 nm and the total P concentrations of the samples were extrapolated from a standard curve constructed using standard solutions of KH_2_PO_4_. Po was calculated from these data as total P minus Pi.

### Root length measurement

For measuring root growth of seedlings on vertical plates, the position of the root tip was marked daily on the plate. At the end of the experiment, the plates were scanned and the incremental changes in root length were measured (LSM Image Browser, Carl Zeiss Microscopy GmbH, Jena, Germany).

### Anthocyanin measurement

Anthocyanins were measured using a pH differential method [66]. Fresh tissue was homogenized at a ratio of 1 mg fresh weight to 10 μl 1% (v/v) acetic acid. The homogenate was centrifuged twice at 12,000 × g for 15 min at 4°C. A 60 μl portion of the final supernatant was diluted with 240 μl of 0.025 M potassium chloride solution, pH 1.0 and another 60 μl aliquot diluted with 240 μl 0.4 M sodium acetate buffer, pH 4.5. The two dilutions, which exhibit the pH-dependent structural transformation of the chromophore of anthocyanins, were equilibrated for 15 min. Sample absorbance was measured at 520 nm and at 700 nm to correct for overall background absorbance from other pigments. The concentration of monomeric anthocyanin pigments was calculated using the formula: Anthocyanin (μmol/g FW) = (A × MW × DF × 1000)/(ε × 1 cm) where: A is the absorbance of diluted sample, MW is the molecular weight of cyanidin-3-glucoside (MW = 449.2), DF is the dilution factor and ε is the molar absorption coefficient for cyanidin-3-glucoside (ε = 26,900 L mol^−1^ cm^−1^) [66].

### Statistical analyses

Unpaired T-tests were done on the qPCR data (GraphPad Quick Calcs, GraphPad Software, Inc., San Jose, CA, USA). Analysis of Variance (ANOVA) followed by Tukey’s Multiple Comparison of Means was done for the root-to-shoot ratio, anthocyanin and Pi measurements, lateral root density, and primary and lateral root lengths (R software, R Foundation for Statistical Computing, Vienna, Austria).

### Availability of supporting data

The data sets supporting the results of this article are included within the article as additional files.

## Additional files

Additional file 1: Figure S1. Plants used in the phosphate (Pi) re-supply experiment. **(A)** Induction of anthocyanin production by Pi deprivation. Red arrows show areas where anthocyanins accumulated. **(B)** Pi concentration in the roots and shoots of control plants and plants re-supplied with Pi for 3 days. Control plants were continuously supplied with 250 μM Pi in nutrient solution for 43 days. For Pi re-supply, plants were continuously provided with 250 μM Pi for 30 d, transferred to solution without Pi for 12 days to deplete internal P pools, and then transferred to a solution containing 250 μM Pi for three days before harvest. Values are means ± S.E. (n = 3 biological replicates). * indicates significantly different means (P <0.05) within a tissue according to Student’s t-test. Additional file 2: Figure S2. Confirmation of *Atpht1* mutant genotypes. Genomic analysis of the homozygous mutants compared to WT **(A)**. Using the left gene primer (LP, LP = PXR in panels **B** to **D** and in the Additional file 11: Table S2, where X = 1, 8 or 9) and the right gene primer (RP, RP = PXF in panels **B** to **D** and in the Additional file 11: Table S2, where X = 1, 8 or 9) produced a gene specific fragment in the wild-type, but not in the knock-out line. Schematic representations of the T-DNA insertion sites in the *Atpht1;1–2* **(B)**, *Atpht1;8* **(C)** and *Atpht1;9–1* **(D)** mutants. Untranslated regions (gray boxes), exons (black boxes), introns (thick black lines), T-DNAs (white boxes) and segments lost during the insertion process (hatched boxes) are shown. DNA sequencing of the fragments amplified using the indicated primers (arrows) confirmed the location of the T-DNA left border (LB) in the genome. The underlined sequence belongs to the T-DNA insert, while the non-underlined sequence belongs to the target gene and identifies the insertion site. Two T-DNAs were tandemly inserted in an inverted orientation in each mutant; hence, the right borders (RB) were presumed to be next to each other. For the *Atpht1;8* mutant, the LB of T-DNA #1 lost 95 bp, while the LB of T-DNA #2 lost 79 bp during insertion. For *Atpht1;9–1* mutant, the LB of T-DNA #1 lost 106 bp, while the LB of T-DNA #2 lost 77 bp during insertion. The seedlings were grown on solid media containing 250 μM Pi for five days before transfer to the same media containing 5 μM Pi for 12 days. Additional file 3: Figure S3. Relative abundance of *AtPHT1;1–2, AtPHT1;8 and AtPHT1;9–1* transcripts in the roots of their respective *Atpht1* knock-out lines (KO) and Col-0 WT described in Additional file 2: Figure S2. RT-PCR **(A)** and qPCR **(B)** analysis of *AtPHT1* transcript abundance in the WT and knock-out mutants. Seedlings were grown in hydroponic medium containing 250 μM Pi for 30 d before transfer to media lacking added Pi for 14 d to maximise *AtPHT1* transcript abundance. Transcript abundance was determined in whole seedlings using gene specific primers (Additional file 11: Table S2) and compared to *AtACT2* transcript abundance as a control for equal loading of cDNA. Transcript abundance of *AtPHT1;1*, *AtPHT1;8* and *AtPHT1;9* was determined in the root of phosphate deprived wild-type plant and their respective KO lines using gene specific primers (Additional file 11: Table S2). The limit of detection for our assay was equivalent to a 40-∆C_t_ value of 30. The residual signal observed for each mutant may represent intact or fragmented transcripts, or may have arisen from spurious products formed in the absence of a *bone fide* target. Values are means ± S.D., n = 3 biological replicates. Additional file 4: Figure S4. Morphological response of WT and *Atpht1* knock-out lines to Pi supply. Seedlings were grown for 5 d on solid medium containing 250 μM Pi before transfer to fresh medium containing either 250 μM Pi (+P) or 5 μM Pi (−P) for 12 d. Additional file 5: Figure S5. Root and shoot fresh weight of *Atpht1* mutant and Col-0 seedlings. These seedlings were those described in Figure 3. Values are means ± S.D. (n = 3 plates of 12 seedlings each). * indicates significantly different means (P <0.05) according to Student’s t-test compared to the WT grown under the same conditions. Additional file 6: Figure S6. Pi depletion of the growth medium by Pi-deprived plants re-supplied with 250 μM Pi. Plants were grown in 150 ml nutrient solution containing 250 μM Pi for 30 d and transferred to solution lacking added Pi for 18 d before a final transfer to solution containing 250 μM Pi. The final nutrient solution was sampled every 30 mins after the last transfer. Values are means ± S.D. (n = 3 boxes with 12 plants each). Trend lines are the best approximation of the initial rates of Pi withdrawal from the solution. The slope of the trend line is given on each graph. The slope (rates) may differ from the values in Table 1, where the average slope from three independent experiments is recorded. Additional file 7: Figure S7. Accumulation of Pi in the WT and *Atpht1* knock-out lines. Time course of Pi accumulation in root **(A)** and shoot **(B)** of WT and *Atpht1* mutant seedlings. Values are means ± S.D. (n = 3 biological replicates with 12 plants each grown at separate times). Data are the same as shown in Figure 4 except that error bars (± S.D.) are shown. Additional file 8: Figure S8. Relative transcript abundance for a panel of 17 Pi-responsive genes in the root **(A)** and shoot **(B)** tissues of *Atpht1;8*. The horizontal black line represents the theoretical detection limit of the qPCR instrument (see Methods). All bars below the line represent transcripts that were below the detection limit. The plants were supplied with 250 μM Pi (sufficient Pi) or no added Pi (no Pi) for 14 d and were the same plants as those described in Figure 5. Values are means ± S.D. (n = 3 biological replicates grown at separate times). * indicates that the 40-∆Ct value in the mutant was significantly different (P <0.05) according to Student’s t-test, compared to the WT grown under the same conditions. Additional file 9: Figure S9. Relative transcript abundance from a panel of 17 Pi-responsive genes in the root **(A)** and shoot **(B)** tissues of Atpht1;9–1. The horizontal black line represents the theoretical detection limit of the qPCR instrument (see Methods). All bars below the line represent transcripts that were below the detection limit. The plants were supplied with 250 μM Pi (sufficient Pi) or no added Pi (no Pi) for 14 d and were the same plants as those described in Figure 5. Values are means ± S.D. (n = 3 biological replicates grown at separate times). * indicates that the 40-∆Ct value in the mutant was significantly different (P <0.05) according to Student’s t-test, compared to the WT grown under the same conditions. Additional file 10: Table S1. Expression levels of AtPHT1 genes derived from microarray experiments using root cells and tissues isolated by fluorescence activated cell sorting of protoplasts from marker lines. Additional file 11: Table S2. Gene specific primers used in the experiment.

## Abbreviations

ANOVA: Analysis of variance
cDNA: Complimentary deoxyribonucleic acid
CTAB: Cetyl trimethylammonium bromide
C_t_: Cycle threshold
Col-0: Columbio-0
d: Days
DF: Dilution factor
DNA: Deoxyribonucleic acid
FW: Fresh weight
MW: Molecular weight
LB: Left-border primer
LP: Left gene-primer
P: Phosphate
Pi: Inorganic phosphate
PHT: Phosphate transporters
qRT-PCR: Quantitative reverse-transciption polymerase chain reaction
RB: Right-border
RP: Right gene primer
SD: Standard deviation
WT: Wild-type
