# Supplementary material for: Arabidopsis PHOSPHATE TRANSPORTER1 genes PHT1;8 and PHT1;9 are involved in root-to-shoot translocation of orthophosphate
Source: BMC Plant Biol. 2014 Nov 27;14:334. doi: 10.1186/s12870-014-0334-z (PMC4252992; doi:10.1186/s12870-014-0334-z)
Supplement: Additional file 1: Figure S1. — Plants used in the phosphate (Pi) re-supply experiment. (A) Induction of anthocyanin production by Pi deprivation. Red arrows show areas where anthocyanins accumulated. (B) Pi concentration in the roots and shoots of control plants and plants re-supplied with Pi for 3 days. Control plants were continuously supplied with 250 μM Pi in nutrient solution for 43 days. For Pi re-supply, plants were continuously provided with 250 μM Pi for 30 d, transferred to solution without Pi for 12 days to deplete internal P pools, and then transferred to a solution containing 250 μM Pi for three days before harvest. Values are means ± S.E. (n = 3 biological replicates). * indicates significantly different means (P <0.05) within a tissue according to Student’s t-test. [file 12870_2014_334_MOESM1_ESM.pdf]

A

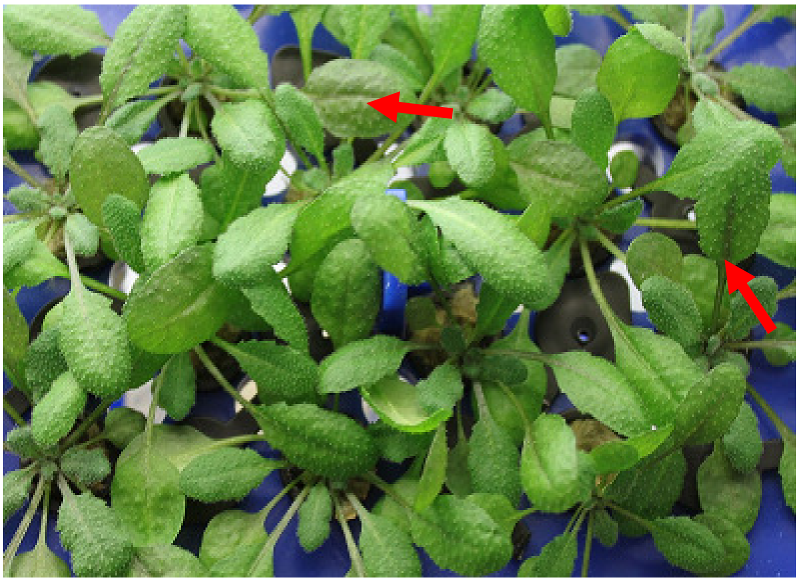

B

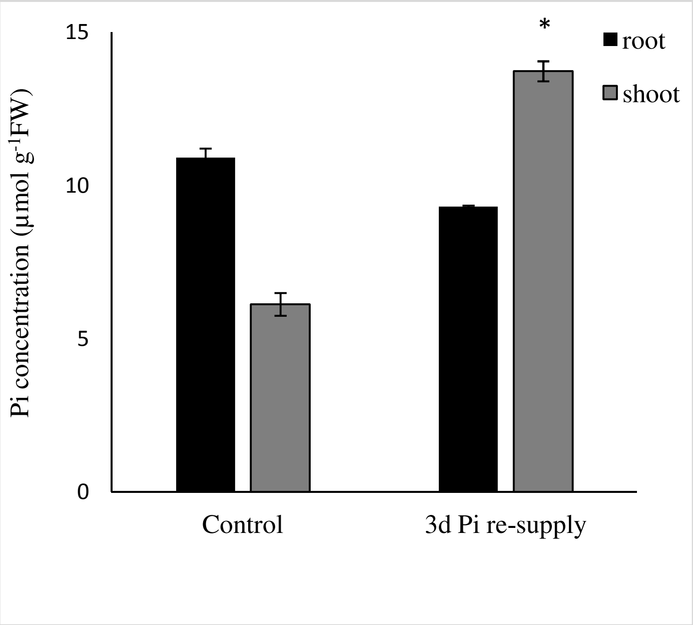

Additional File: Figure S1. Plants used in the phosphate (Pi) re-supply experiment. (A) Induction of anthocyanin production by Pi deprivation. Red arrows show areas where anthocyanins accumulated. (B) Pi concentration in the roots and shoots of control plants and plants re-supplied with Pi for 3 days. Control plants were continuously supplied with 250 μM Pi in nutrient solution for 43 days. For Pi re-supply, plants were continuously provided with 250 μM Pi for 30 d, transferred to solution without Pi for 12 days to deplete internal P pools, and then transferred to a solution containing 250 μM Pi for three days before harvest. Values are means ± S.E. (n = 3 biological replicates). * indicates significantly different means ($P < 0.05$) within a tissue according to Student's t-test.
