# Supplementary material for: Arabidopsis PHOSPHATE TRANSPORTER1 genes PHT1;8 and PHT1;9 are involved in root-to-shoot translocation of orthophosphate
Source: BMC Plant Biol. 2014 Nov 27;14:334. doi: 10.1186/s12870-014-0334-z (PMC4252992; doi:10.1186/s12870-014-0334-z)
Supplement: Additional file 2: Figure S2. — Confirmation of Atpht1 mutant genotypes. Genomic analysis of the homozygous mutants compared to WT (A). Using the left gene primer (LP, LP = PXR in panels B to D and in the Additional file 11: Table S2, where X = 1, 8 or 9) and the right gene primer (RP, RP = PXF in panels B to D and in the Additional file 11: Table S2, where X = 1, 8 or 9) produced a gene specific fragment in the wild-type, but not in the knock-out line. Schematic representations of the T-DNA insertion sites in the Atpht1;1–2 (B), Atpht1;8 (C) and Atpht1;9–1 (D) mutants. Untranslated regions (gray boxes), exons (black boxes), introns (thick black lines), T-DNAs (white boxes) and segments lost during the insertion process (hatched boxes) are shown. DNA sequencing of the fragments amplified using the indicated primers (arrows) confirmed the location of the T-DNA left border (LB) in the genome. The underlined sequence belongs to the T-DNA insert, while the non-underlined sequence belongs to the target gene and identifies the insertion site. Two T-DNAs were tandemly inserted in an inverted orientation in each mutant; hence, the right borders (RB) were presumed to be next to each other. For the Atpht1;8 mutant, the LB of T-DNA #1 lost 95 bp, while the LB of T-DNA #2 lost 79 bp during insertion. For Atpht1;9–1 mutant, the LB of T-DNA #1 lost 106 bp, while the LB of T-DNA #2 lost 77 bp during insertion. The seedlings were grown on solid media containing 250 μM Pi for five days before transfer to the same media containing 5 μM Pi for 12 days. [file 12870_2014_334_MOESM2_ESM.pdf]

A

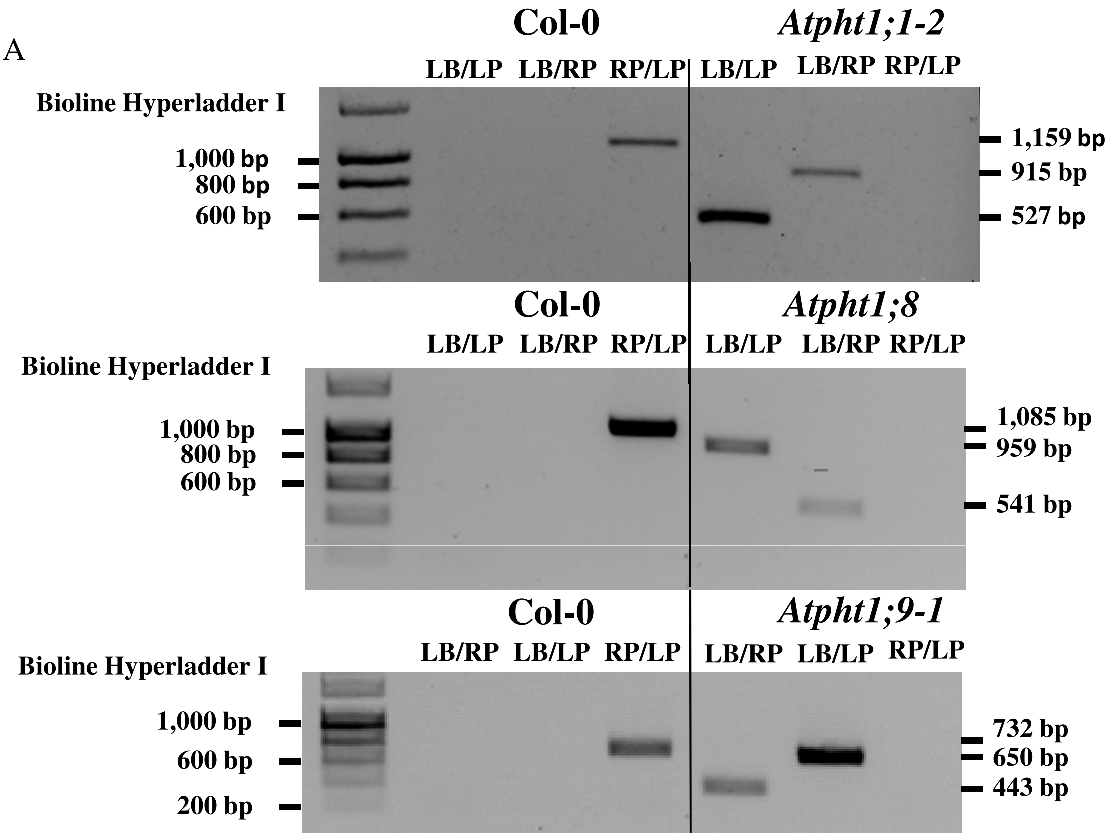

Col-0
Atpht1;1-2
LB/LP LB/RP RP/LP LB/LP LB/RP RP/LP
Bioline Hyperladder I
1,000 bp
800 bp
600 bp
1,159 bp
915 bp
527 bp
Col-0
Atpht1;8
LB/LP LB/RP RP/LP LB/LP LB/RP RP/LP
Bioline Hyperladder I
1,000 bp
800 bp
600 bp
1,085 bp
959 bp
541 bp
Col-0
Atpht1;9-1
LB/RP LB/LP RP/LP LB/RP LB/LP RP/LP
Bioline Hyperladder I
1,000 bp
600 bp
200 bp
732 bp
650 bp
443 bp

B *AtPHT1;1-2*

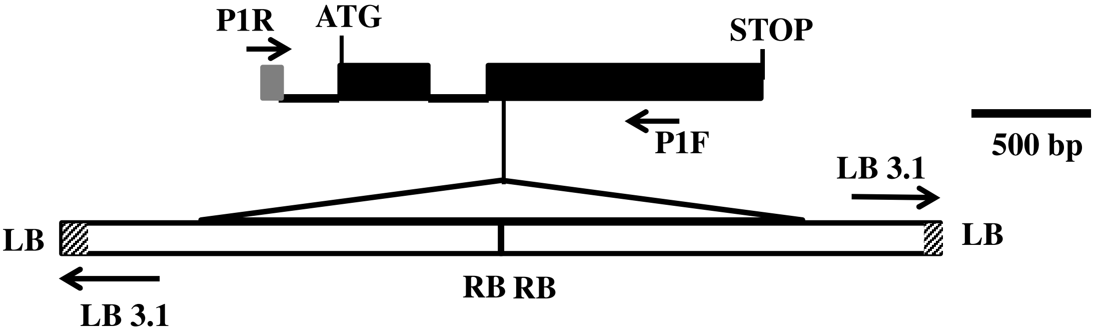

P1R--LB3.1 amplicon:

5’-TACGCAAACAAGAAGACCCGTGGGGCTTTCATCGCAGCTGTCTTCGCCATGCAAGGTGTCGGTATCT
TGGCTGGAGGTTTCGTGGCACTCGCAGTATCTTCTATATTCGACAAAAAGTTCCCAGCTCCAACATATGC
AGTAAACAGGGCCCTCTCAACGCCTCCTCAAGTTGACTACATTTGGCGAATCATCGTCATGTTTGGTGCT
TTACCCGCAGCTTTGACTTACTACTGGCGTATGAAGATGCCTGAAACTGCCCGTTACACCGCTTTGGTTGC
CAAGAACATCAAACAAGCCACAGCCGACATGTCCAAGGTCTTACAAACAGATATCGAGCTTGAGGAAAG
GGTGGAGGATGACGTCAAAGACCCCAAACAAAACTATGGCTTGCTCAGGGCCAGGCGGTGAAGGGCAA
TCAGCTGTTGCCCGTCTCACTGGTGAAAAGAAAAACCCACCCCCCAGTACATTAAAAACGTCCGCAATGT
GTTATTAAGTTTTTAAGCGTCAATGTGTTAT-3’

P1F--LB3.1 amplicon:

5’ CTGTTGCCCGTCTCACTGGTGAAAAGAAAAACCACCCCAGTACATTAAAAACGTCCGCAATGTGTTAT
TAAGTTGTCTAAGCGTCAATTTGTTTACACCACAATATCTCCTTGGAACTACCTCCACATGGTTTTTGCTT
GACATTGCCTTCTACAGCCAAAACTTGTTCCAGAAGGATATTTTCTCGGCCATCGGATGGATCCCAAAGG
CAGCCACCATGAACGCCACCCATGAGGTTTTCAGGATTGCTAGGGCTCAGACTCTTATCGCCCTTTGCAG
TACAGTCCCAGGCTACTGGTTCACAGTTGCGTTTATTGATACCATTG-3’

C

AtPHT1;8

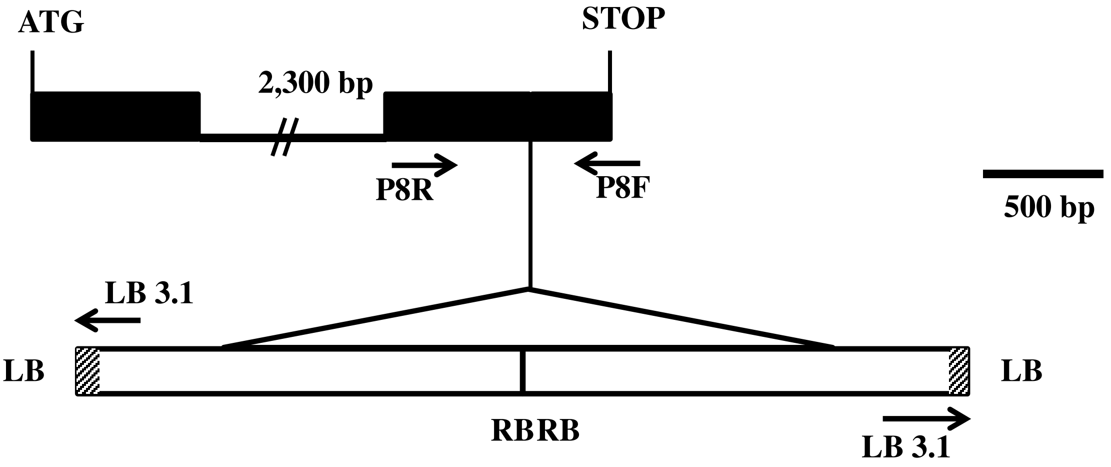

P8F--LB3.1 amplicon:

5'-GACATGCAAAGAGTCATGTCAAGATCTCATATCTCCGACGAAGCCACGACGGATCCACCTCCTCC
TCCGCCGCCACCTTCCTACAAACTCTTCTCCCGCTGCTTCTTCCGCCTCCATGGTCGCGACCTCTTCGCAG
CCTCCTTTAATTGGTTCCTAGTAGACATTGTCTTCTACACAAGCAACCTCCTCCTGTCCCATATCTTCAGT
CATTACTCCAAGAAACCTTCCACTGCAGAAAACGTCTACGACGCAGCCTTTGAAGTGGCGGAATTAGG
AGCCATCATCGCTGCTTGCTCCACCATTCCCGGTTATTGGTTCACAGTTTACTTCATCGACAAGATAGGT
CGTGTCAAAATCCAGATTATGGGGTTTTTCTTCATGGCCGTTATTTATTTAGTCGCTGGTATTCCGTACA
GTTGGTATTGGTCAAAGCACGAGCATAATAATAAAGGCTTCATGGTTCTCTACGGTTTGGTTTTCTTCTT
TTGCAATTTTGGTCCCAACACTACAACTTTTATTATTCCCGCAGAGCATTTCCCGGCTAGGTTTAGGCAA
ATTGACGCTTAGACAACTTAATAACACATTGCGGACGTTTTTAATGTACTGGGGTGGTTTTTCTTTTCAC
CAGTGAGACGGGCAACAGCTGATTGCCCTTCACCGCCTGGCCCTGAGAGAGTTGCAGCAAGCGGTCCA
CGCTGGTTTGCCCCAGCAGGCGAAAATCCTGTTT-3'

P8R--LB3.1 amplicon:

5'-AAACAAAATCTAGAAAAGATTAGCAGAAATATACAATTGTATGAACTTGAACTGATCATGTGTTCA
ATATGTCGAGATCATCATCGTGTCTGAAGCAAAGTGCTCACGGAGGATTGCCCGTCAACAATCTGTGGC
TCATCTTCAGACTCAGCATTGTTATCTTGGTCATGCTCGTTCTCCTCTAACGATCTTCCCATAGTTTCCTT
GGTGAAGAAATACGTCACCAAGATTCCAGCAATGCAAACGCCACCAAGAATCAAGAAAGCAATTCTCA
TACGGTTTACTTCGGGATAAATCTGGTTTTTATCATCGCTTTCCATCTTCTTGGTAGCCCACAAGAACCC
AACAGTGCCCACAATAGCCCCAAGCTTACCCGCGGCACCAGAGATCCCGTGACAAGTATATATTGTGGT
GTAAACAAATTGACGCTTAGACAACTTAATAACACATTGCGGACGTTTTTAATGTACTGGGGTGGTTTT
TCTTTTCACCAGTGAGACGGGCAACAGCTGATTGCCCTTCACCGCCTGGCCCTGAGAGAGTTGCAGCAA
GCGGTCCACGCTGGTTTGCCCCAGCAGGCGAAAATCCTGTTTAGGGGGGGGGGTTCCGGAAAAAAAGGG
GGGGC-3'

D *AtPHT1;9-1*

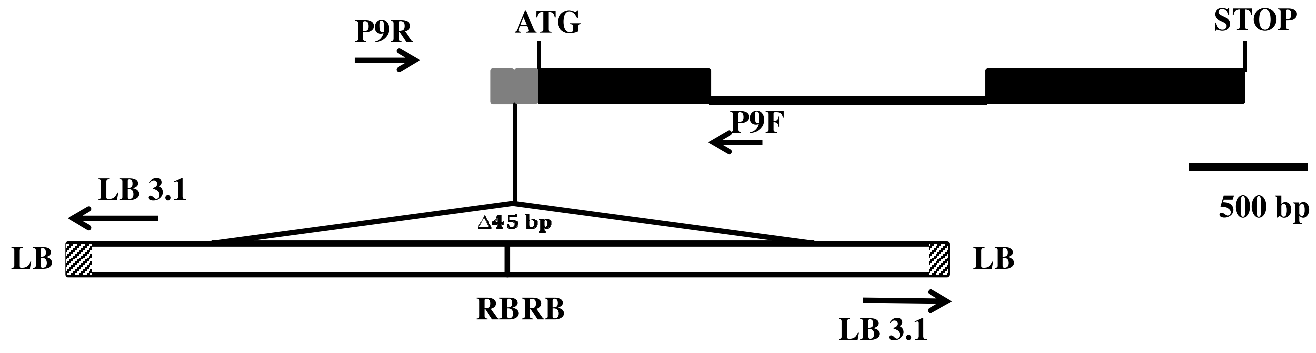

P9R--LB3.1 amplicon:

5’-TCACATCTTACAACTAAAACTCTTTGTACCAAAGAGGGAAATTTAAAACTTATAATCCAAATTTTATA
AATAAAGTTGTCAAAGTCAACTAAAGAAGTGGACTCGATCCAGGGAGGTGTTGTGTTCCTCACAGTCAA
ACCAAAGCATAGACAACTTAATAACACATTGCGGACGTTTTTAATGTACTGGGGTGGTTTTTCTTTTCAC
CAGTGAGACGGGCAACAGCTGATTGCCCTTCACCGCCTGGCCCTGAGAGAGTTGCAGCAAGCGGTCCAC
GCTGGTTTGCCCCAGCAGGCGAAAATCCTGTTTGATGGTGGTTCCGA-3’

P9F--LB3.1 amplicon:

5’-TCGGAACCACCATCAAACAGGATTTTCGCCTGCTGGGGCAAACCAGCGTGGACCGCTTGCTGCAACT
CTCTCAGGGCCAGGCGGTGAAGGGCAATCAGCTGTTGCCCGTCTCACTGGTGAAAAGAAAAACCACCC
CAGTACATTAAAAACGTCCGCAATGTGTTATTAAGTTGTCTAAGCGTCAATTTGTTTACACCACAATCTG
TCAAATGAAAAATACAAAGACTTCTATTGTTCTCATTCTCCGTATCTTGAAAAAATTCTTAAAAAATCG
ACGAAACGATGCCGGAGTTAAGTTTATTATCGGCGTTAGACGCAGCGAGGATACAGTGGTACCATTTCA
AAGCGATAATAGTCGCCGGAATGGGACTTTTCACCGACGCATACGATCTTTTCTGTATAGCTCCGATCA
TGAAAATGATAAGTCAAATATATTACCACAAAGACTCAATCGGAACCGCTCTTCTCTCAACTTCTTACG
CAATCGCTCTTCTTGGCACCGCGTTAGGTCAGCTCATCTTTGGTTACTTAGGTGACCGAGTCGGACGTCG
TAAAGTCTATGGACTTAGTCTCTTGATCATGGTGTTTAGCTCCTTTGGTTGTGGCTTCTCTGTTTGCACGA
CTCGACGTTCTTGTGTTATGGTTAGTCT-3’

Additional File: Figure S2. Confirmation of *Atpht1* mutant genotypes. Genomic analysis of the homozygous mutants compared to WT (A). Using the left gene primer (LP, LP = PXR in panels B to D and in the Additional File: Table S2, where X = 1, 8 or 9) and the right gene primer (RP, RP = PXF in panels B to D and in the Additional File: Table S2, where X = 1, 8 or 9) produced a gene specific fragment in the wild-type, but not in the knock-out line. Schematic representations of the T-DNA insertion sites in the *Atpht1;1-2* (B), *Atpht1;8* (C) and *Atpht1;9-1* (D) mutants. Untranslated regions (gray boxes), exons (black boxes), introns (thick black lines), T-DNAs (white boxes) and segments lost during the insertion process (hatched boxes) are shown. DNA sequencing of the fragments amplified using the indicated primers (arrows) confirmed the location of the T-DNA left border (LB) in the genome. The underlined sequence belongs to the T-DNA insert, while the non-underlined sequence belongs to the target gene and identifies the insertion site. Two T-DNAs were tandemly inserted in an inverted orientation in each mutant; hence, the right borders (RB) were presumed to be next to each other. For the *Atpht1;8* mutant, the LB of T-DNA #1 lost 95 bp, while the LB of T-DNA #2 lost 79 bp during insertion. For *Atpht1;9-1* mutant, the LB of T-DNA #1 lost 106 bp, while the LB of T-DNA #2 lost 77 bp during insertion. The seedlings were grown on solid media containing 250 μM Pi for five days before transfer to the same media containing 5 μM Pi for 12 days.
