# Supplementary material for: Arabidopsis PHOSPHATE TRANSPORTER1 genes PHT1;8 and PHT1;9 are involved in root-to-shoot translocation of orthophosphate
Source: BMC Plant Biol. 2014 Nov 27;14:334. doi: 10.1186/s12870-014-0334-z (PMC4252992; doi:10.1186/s12870-014-0334-z)
Supplement: Additional file 3: Figure S3. — Relative abundance of AtPHT1;1–2, AtPHT1;8 and AtPHT1;9–1 transcripts in the roots of their respective Atpht1 knock-out lines (KO) and Col-0 WT described in Additional file 2: Figure S2. RT-PCR (A) and qPCR (B) analysis of AtPHT1 transcript abundance in the WT and knock-out mutants. Seedlings were grown in hydroponic medium containing 250 μM Pi for 30 d before transfer to media lacking added Pi for 14 d to maximise AtPHT1 transcript abundance. Transcript abundance was determined in whole seedlings using gene specific primers (Additional file 11: Table S2) and compared to AtACT2 transcript abundance as a control for equal loading of cDNA. Transcript abundance of AtPHT1;1, AtPHT1;8 and AtPHT1;9 was determined in the root of phosphate deprived wild-type plant and their respective KO lines using gene specific primers (Additional file 11: Table S2). The limit of detection for our assay was equivalent to a 40-∆Ct value of 30. The residual signal observed for each mutant may represent intact or fragmented transcripts, or may have arisen from spurious products formed in the absence of a bone fide target. Values are means ± S.D., n = 3 biological replicates. [file 12870_2014_334_MOESM3_ESM.pdf]

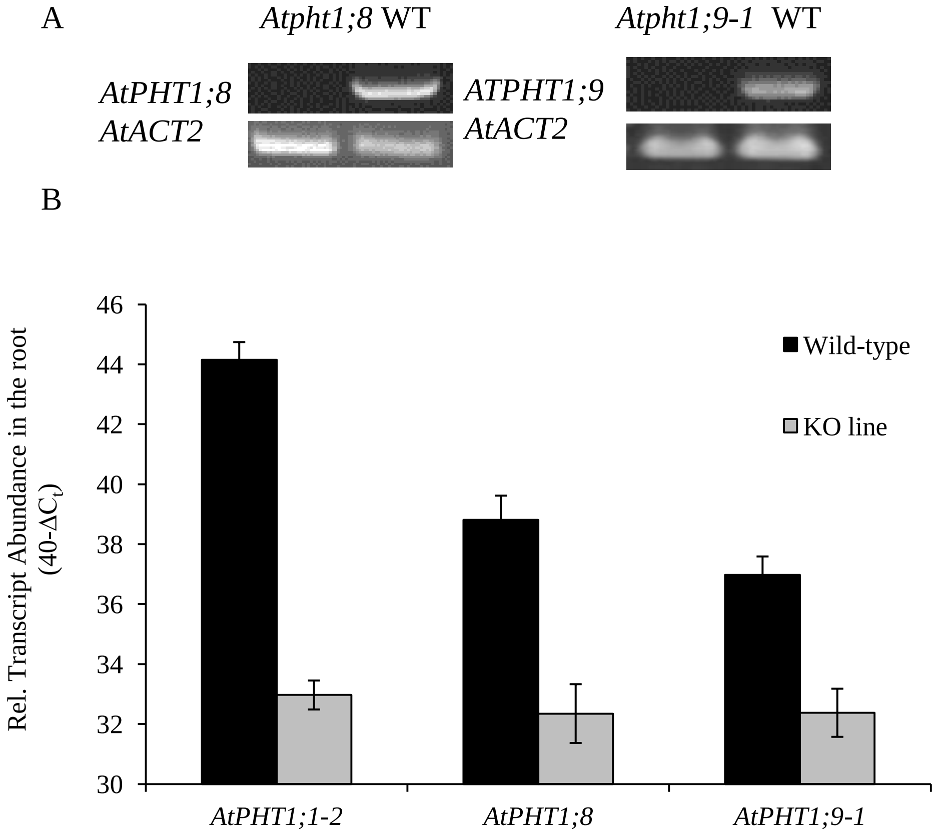

Additional File: Figure S3. Relative abundance of *AtPHT1;1-2, AtPHT1;8 and AtPHT1;9-1* transcripts in the roots of their respective *Atpht1* knock-out lines (KO) and Col-0 WT described in Additional File: Figure S2. RT-PCR (A) and qPCR (B) analysis of *AtPHT1* transcript abundance in the WT and knock-out mutants. Seedlings were grown in hydroponic medium containing 250 µM Pi for 30 d before transfer to media lacking added Pi for 14 d to maximise *AtPHT1* transcript abundance. Transcript abundance was determined in whole seedlings using gene specific primers (Additional File: Table S2) and compared to *AtACT2* transcript abundance as a control for equal loading of cDNA. Transcript abundance of *AtPHT1;1*, *AtPHT1;8* and *AtPHT1;9* was determined in the root of phosphate deprived wild-type plant and their respective KO lines using gene specific primers (Additional File: Table S2). The limit of detection for our assay was equivalent to a 40-ΔC_t value of 30. The residual signal observed for each mutant may represent intact or fragmented transcripts, or may have arisen from spurious products formed in the absence of a *bone fide* target. Values are means ± S.D., n = 3 biological replicates.
