# Supplementary material for: Arabidopsis PHOSPHATE TRANSPORTER1 genes PHT1;8 and PHT1;9 are involved in root-to-shoot translocation of orthophosphate
Source: BMC Plant Biol. 2014 Nov 27;14:334. doi: 10.1186/s12870-014-0334-z (PMC4252992; doi:10.1186/s12870-014-0334-z)
Supplement: Additional file 4: Figure S4. — Morphological response of WT and Atpht1 knock-out lines to Pi supply. Seedlings were grown for 5 d on solid medium containing 250 μM Pi before transfer to fresh medium containing either 250 μM Pi (+P) or 5 μM Pi (−P) for 12 d. [file 12870_2014_334_MOESM4_ESM.pdf]

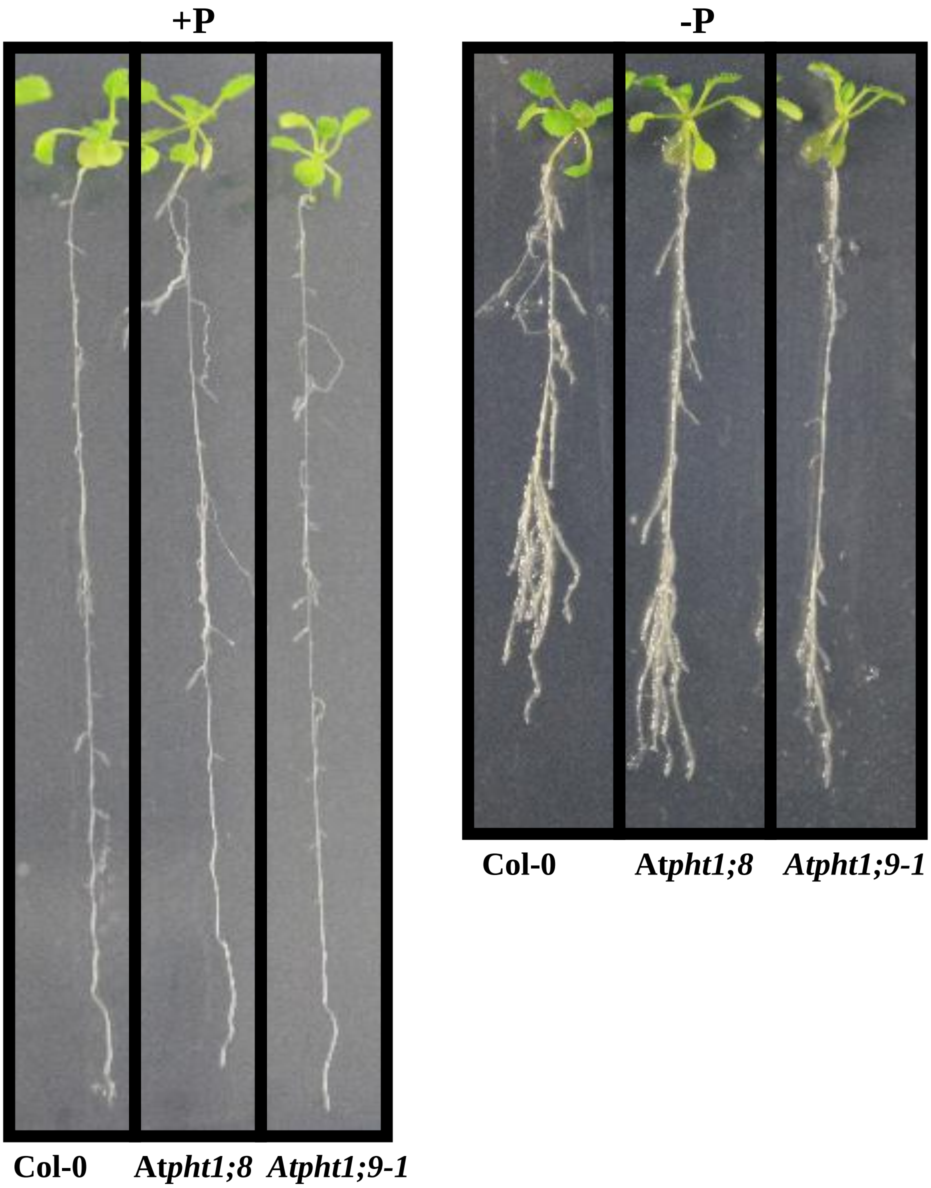

Additional File: Figure S4. Morphological response of WT and *Atpht1* knock-out lines to Pi supply. Seedlings were grown for 5 d on solid medium containing 250 µM Pi before transfer to fresh medium containing either 250 µM Pi (+P) or 5 µM Pi (-P) for 12 d.
