# Supplementary material for: Arabidopsis PHOSPHATE TRANSPORTER1 genes PHT1;8 and PHT1;9 are involved in root-to-shoot translocation of orthophosphate
Source: BMC Plant Biol. 2014 Nov 27;14:334. doi: 10.1186/s12870-014-0334-z (PMC4252992; doi:10.1186/s12870-014-0334-z)
Supplement: Additional file 5: Figure S5. — Root and shoot fresh weight of Atpht1 mutant and Col-0 seedlings. These seedlings were those described in Figure 3. Values are means ± S.D. (n = 3 plates of 12 seedlings each). * indicates significantly different means (P <0.05) according to Student’s t-test compared to the WT grown under the same conditions. [file 12870_2014_334_MOESM5_ESM.pdf]

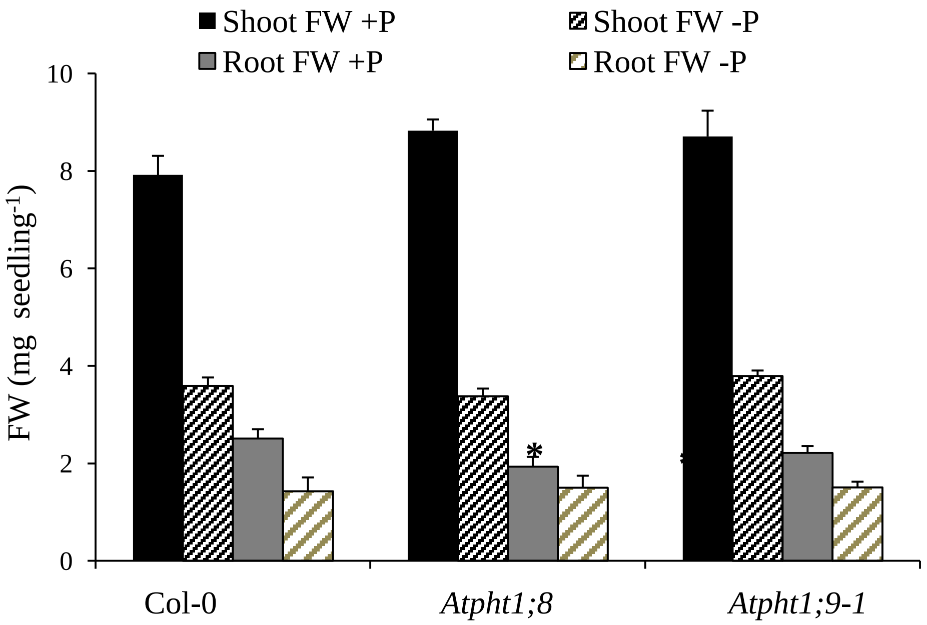

Additional File: Figure S5. Root and shoot fresh weight of *Atpht1* mutant and Col-0 seedlings. These seedlings were those described in Figure 3. Values are means ± S.D. (n = 3 plates of 12 seedlings each). * indicates significantly different means ($P < 0.05$) according to Student's t-test compared to the WT grown under the same conditions.
