# Supplementary material for: Arabidopsis PHOSPHATE TRANSPORTER1 genes PHT1;8 and PHT1;9 are involved in root-to-shoot translocation of orthophosphate
Source: BMC Plant Biol. 2014 Nov 27;14:334. doi: 10.1186/s12870-014-0334-z (PMC4252992; doi:10.1186/s12870-014-0334-z)
Supplement: Additional file 6: Figure S6. — Pi depletion of the growth medium by Pi-deprived plants re-supplied with 250 μM Pi. Plants were grown in 150 ml nutrient solution containing 250 μM Pi for 30 d and transferred to solution lacking added Pi for 18 d before a final transfer to solution containing 250 μM Pi. The final nutrient solution was sampled every 30 mins after the last transfer. Values are means ± S.D. (n = 3 boxes with 12 plants each). Trend lines are the best approximation of the initial rates of Pi withdrawal from the solution. The slope of the trend line is given on each graph. The slope (rates) may differ from the values in Table 1, where the average slope from three independent experiments is recorded. [file 12870_2014_334_MOESM6_ESM.pdf]

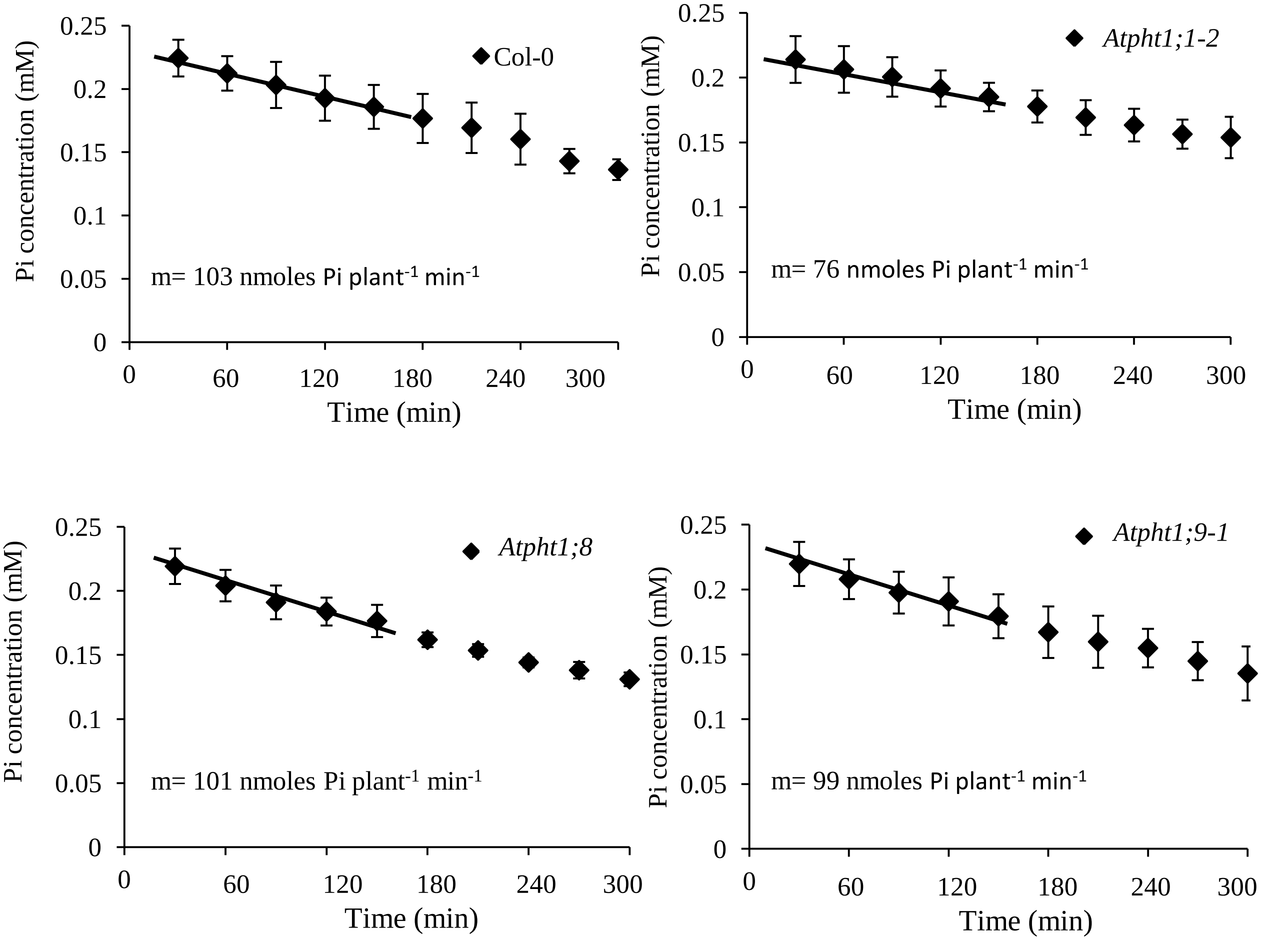

Additional File: Figure S6. Pi depletion of the growth medium by Pi-deprived plants re-supplied with 250 µM Pi. Plants were grown in 150 ml nutrient solution containing 250 µM Pi for 30 d and transferred to solution lacking added Pi for 18 d before a final transfer to solution containing 250 µM Pi. The final nutrient solution was sampled every 30 mins after the last transfer. Values are means ± S.D. (n = 3 boxes with 12 plants each). Trend lines are the best approximation of the initial rates of Pi withdrawal from the solution. The slope of the trend line is given on each graph. The slope (rates) may differ from the values in Table 1, where the average slope from three independent experiments is recorded.
