# Supplementary material for: Arabidopsis PHOSPHATE TRANSPORTER1 genes PHT1;8 and PHT1;9 are involved in root-to-shoot translocation of orthophosphate
Source: BMC Plant Biol. 2014 Nov 27;14:334. doi: 10.1186/s12870-014-0334-z (PMC4252992; doi:10.1186/s12870-014-0334-z)
Supplement: Additional file 7: Figure S7. — Accumulation of Pi in the WT and Atpht1 knock-out lines. Time course of Pi accumulation in root (A) and shoot (B) of WT and Atpht1 mutant seedlings. Values are means ± S.D. (n = 3 biological replicates with 12 plants each grown at separate times). Data are the same as shown in Figure 4 except that error bars (± S.D.) are shown. [file 12870_2014_334_MOESM7_ESM.pdf]

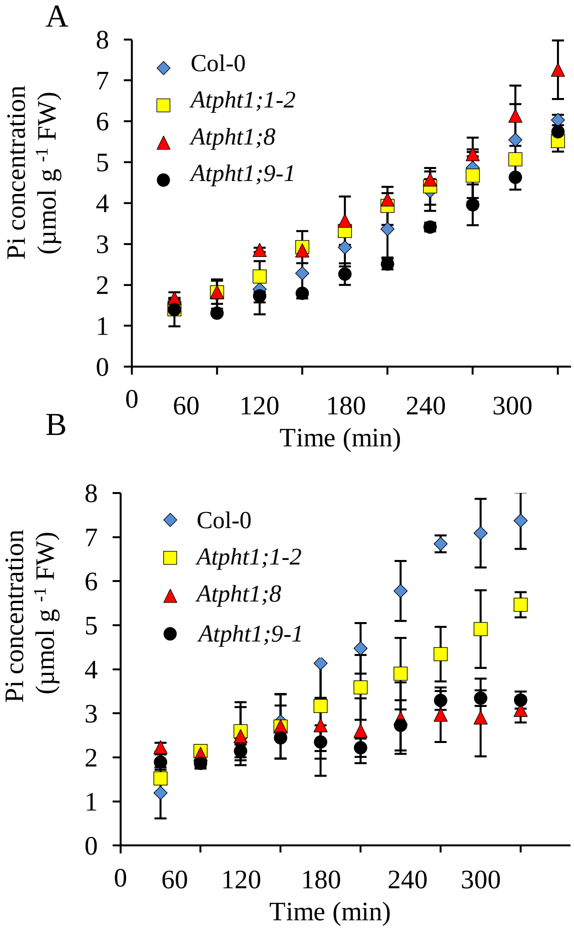

Additional File: Figure S7. Accumulation of Pi in the WT and *Atpht1* knock-out lines. Time course of Pi accumulation in root (A) and shoot (B) of WT and *Atpht1* mutant seedlings. Values are means ± S.D. (n = 3 biological replicates with 12 plants each grown at separate times). Data are the same as shown in Figure 4 except that error bars (± S.D.) are shown.
