# Supplementary material for: Arabidopsis PHOSPHATE TRANSPORTER1 genes PHT1;8 and PHT1;9 are involved in root-to-shoot translocation of orthophosphate
Source: BMC Plant Biol. 2014 Nov 27;14:334. doi: 10.1186/s12870-014-0334-z (PMC4252992; doi:10.1186/s12870-014-0334-z)
Supplement: Additional file 8: Figure S8. — Relative transcript abundance for a panel of 17 Pi-responsive genes in the root (A) and shoot (B) tissues of Atpht1;8. The horizontal black line represents the theoretical detection limit of the qPCR instrument (see Methods). All bars below the line represent transcripts that were below the detection limit. The plants were supplied with 250 μM Pi (sufficient Pi) or no added Pi (no Pi) for 14 d and were the same plants as those described in Figure 5. Values are means ± S.D. (n = 3 biological replicates grown at separate times). * indicates that the 40-∆Ct value in the mutant was significantly different (P <0.05) according to Student’s t-test, compared to the WT grown under the same conditions. [file 12870_2014_334_MOESM8_ESM.pdf]

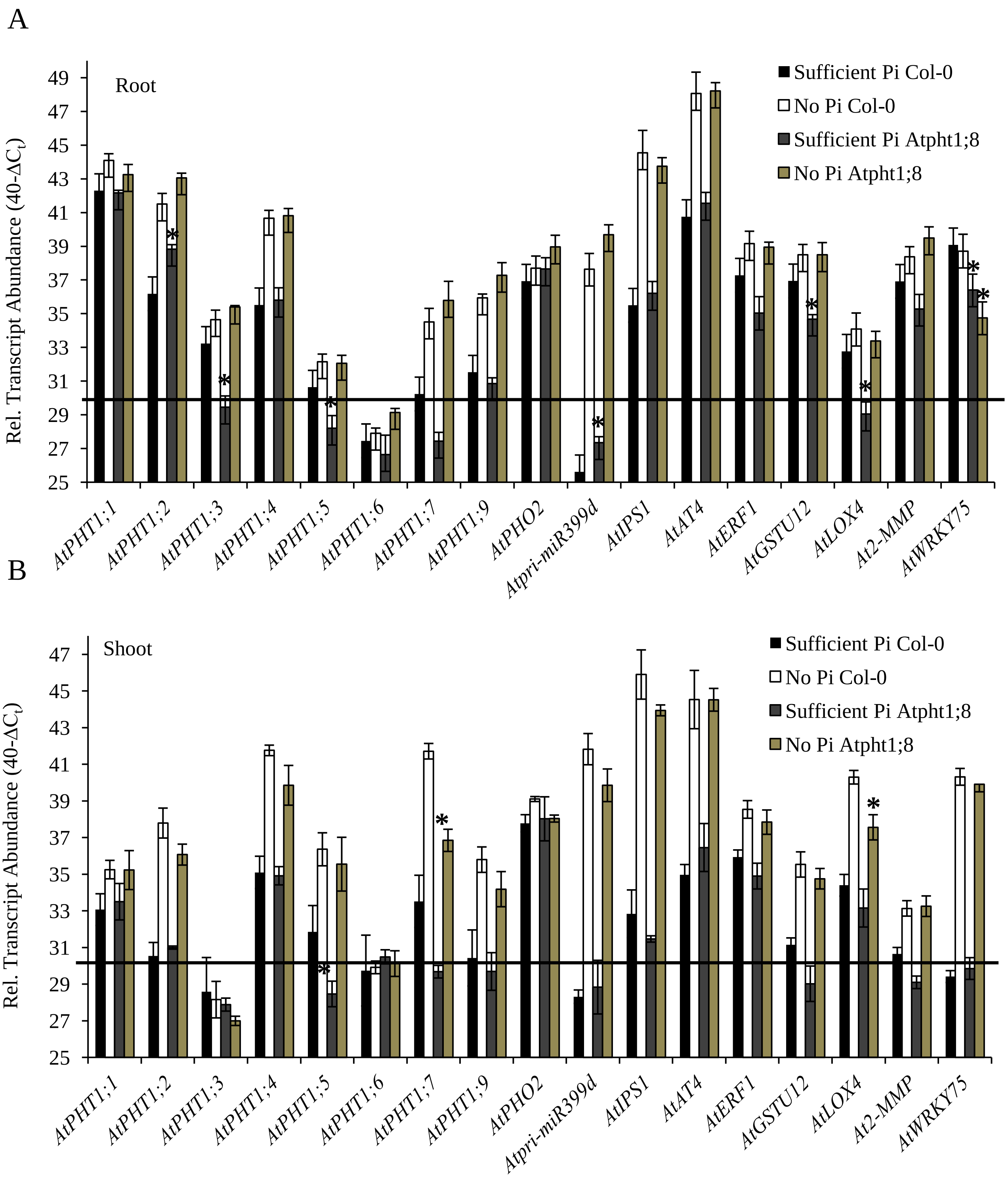

Additional File: Figure S8. Relative transcript abundance for a panel of 17 Pi-responsive genes in the root (A) and shoot (B) tissues of *Atpht1;8*. The horizontal black line represents the theoretical detection limit of the qPCR instrument (see Materials and Methods). All bars below the line represent transcripts that were below the detection limit. The plants were supplied with 250 µM Pi (sufficient Pi) or no added Pi (no Pi) for 14 d and were the same plants as those described in Figure 5. Values are means ± S.D. (n = 3 biological replicates grown at separate times). * indicates that the 40-ΔCt value in the mutant was significantly different ($P < 0.05$) according to Student's t-test, compared to the WT grown under the same conditions.
